# Oxygen Isotope Variability within *Nautilus* Shell Growth Bands

**DOI:** 10.1371/journal.pone.0153890

**Published:** 2016-04-21

**Authors:** Benjamin J. Linzmeier, Reinhard Kozdon, Shanan E. Peters, John W. Valley

**Affiliations:** 1 Department of Geoscience, University of Wisconsin-Madison, Madison, Wisconsin, United States of America; 2 Lamont-Doherty Earth Observatory, Columbia University, Palisades, New York, United States of America; 3 WiscSIMS, Department of Geoscience, University of Wisconsin-Madison, Madison, Wisconsin, United States of America; Naturhistoriska riksmuseet, SWEDEN

## Abstract

*Nautilus* is often used as an analogue for the ecology and behavior of extinct externally shelled cephalopods. *Nautilus* shell grows quickly, has internal growth banding, and is widely believed to precipitate aragonite in oxygen isotope equilibrium with seawater. Pieces of shell from a wild-caught *Nautilus macromphalus* from New Caledonia and from a *Nautilus belauensis* reared in an aquarium were cast in epoxy, polished, and then imaged. Growth bands were visible in the outer prismatic layer of both shells. The thicknesses of the bands are consistent with previously reported daily growth rates measured in aquarium reared individuals. *In situ* analysis of oxygen isotope ratios using secondary ion mass spectrometry (SIMS) with 10 μm beam-spot size reveals inter- and intra-band δ^18^O variation. In the wild-caught sample, a traverse crosscutting 45 growth bands yielded δ^18^O values ranging 2.5‰, from +0.9 to -1.6 ‰ (VPDB), a range that is larger than that observed in many serial sampling of entire shells by conventional methods. The maximum range within a single band (~32 μm) was 1.5‰, and 27 out of 41 bands had a range larger than instrumental precision (±2 SD = 0.6‰). The results from the wild individual suggest depth migration is recorded by the shell, but are not consistent with a simple sinusoidal, diurnal depth change pattern. To create the observed range of δ^18^O, however, this *Nautilus* must have traversed a temperature gradient of at least ~12°C, corresponding to approximately 400 m depth change. Isotopic variation was also measured in the aquarium-reared sample, but the pattern within and between bands likely reflects evaporative enrichment arising from a weekly cycle of refill and replacement of the aquarium water. Overall, this work suggests that depth migration behavior in ancient nektonic mollusks could be elucidated by SIMS analysis across individual growth bands.

## Introduction

Stable carbon and oxygen isotope analyses have been used to understand living *Nautilus* ecology [1–6] and to infer the ecology of extinct externally shelled cephalopods [7–11]. Data from aquarium rearing suggest that *Nautilus*, like many mollusks, precipitate their shells in oxygen isotope equilibrium with ambient temperature and seawater δ^18^O [12,13]. In the modern ocean, both the temperature and the oxygen isotope ratio of water vary with depth and seasons; however, strong seasonality is not present in low-latitude *Nautilus* habitat [14,15]. Many authors have argued that δ^18^O change in large samples of wild-caught *Nautilus* shell are a change in average living depth through time [1,2,8,11,12].

*Nautilus* are active swimmers that traverse hundreds of meters water depth during the day within the steep forereef environment in search of food and to avoid predators [16–19]. Photocapture traps have demonstrated that whole populations migrate several hundred meters through the water column daily [20]. Individual *Nautilus* have been tracked with remote telemetry crossing depths from >200 m to <50 m, spanning a range in water temperature of over 3°C, within individual days [17–19]. If shell precipitation is continuous across all depths in a migrating *Nautilus*, a change in δ^18^O should be observed within a single day’s shell growth in a wild-caught individual.

**Fig 1:**
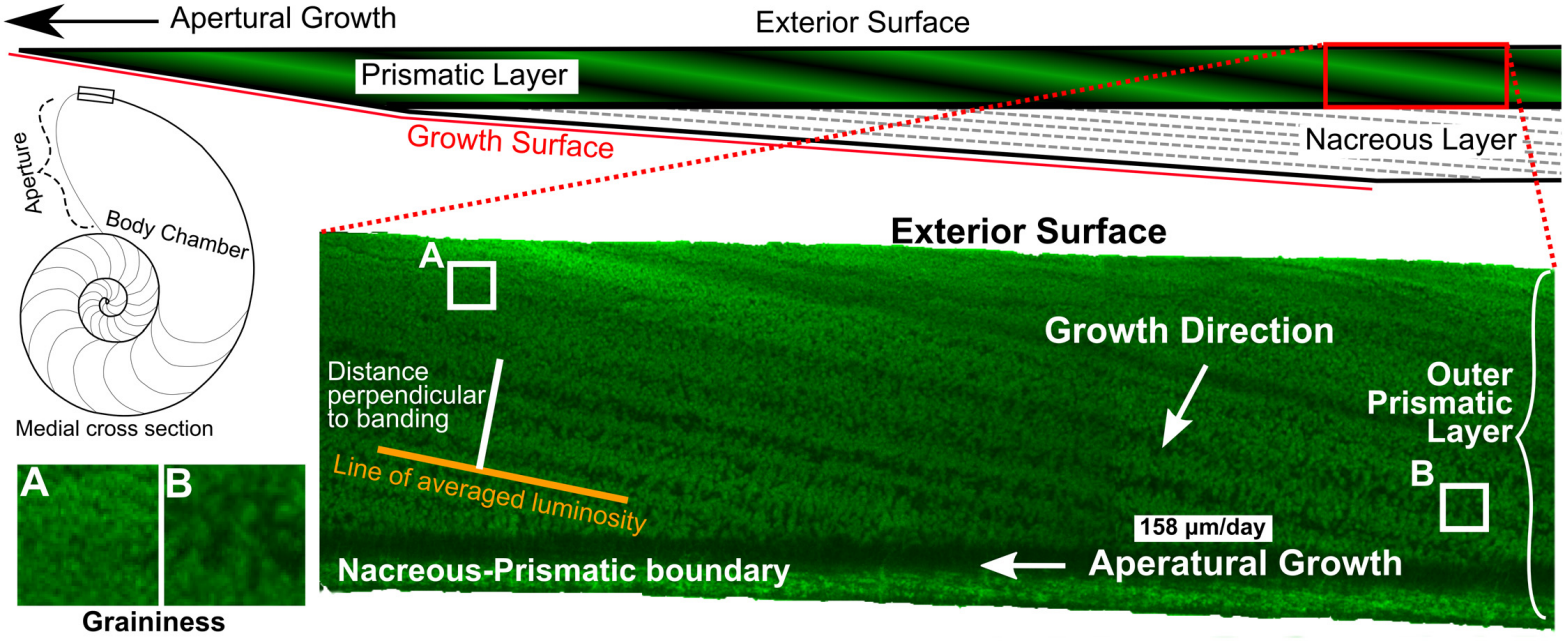
Schematic illustrations of a cut through the medial plane of a *Nautilus* and full thickness shell wall at the aperture and CLFM image of *Nautilus* shell showing inclined banding visible by CLFM in the outer prismatic layer relative to actively growing surface of the shell. SIMS measurements in this study were only from the outer prismatic layer of shell and no analyses are reported from the nacreous layer because growth banding in the nacreous layer is too thin for depth migration to be subsampled by 10-μm SIMS spots. The CLFM image shows only the outer prismatic layer of the *Nautilus* shell. Growth bands were formed in the layer parallel to the growth surface and are likely expressed due to differences in the proportion of aragonite to intercrystalline organic matter [22]. Growth bands extend from the exterior surface of the shell to the nacreous-prismatic boundary and are inclined by ~11° to the exterior surface. Growth rates in literature (averaging 158 μm/day as represented by the scale bar) are typically reported as apertural growth per day, which corresponds to the distance along the exterior surface in the figure [21]. There are three major patterns within growth bands: 1) There is less contrast between the light and dark portions of bands near the exterior surface. 2) Near the exterior surface spots of brightly luminescing aragonite are small and surrounded by small spots of less luminescing aragonite (less graininess) (A). 3) Near the nacreous-prismatic boundary there are larger spots of brightly luminescing aragonite surrounded by continuous areas that do not luminesce as brightly (B). The intensity of fluorescence was measured along the distance perpendicular to banding by averaging lines of pixels parallel to growth banding.

Most stable isotope studies to date have used phosphoric acid digestion coupled with gas-source mass spectrometry and therefore used relatively large volume samples (>40 μg of powder) that were collected by drilling or mechanical removal of chips (e.g. [1]). Thus, traditional methods must average multiple days of growth when extracting a sample, which precludes the detection of all short-term depth migration behavior and instead provides an average temperature for the duration of growth sampled. One study by Oba et al. [2] used finer scale physical sampling within septa that grow to full thickness over ~2 months [21]. They planed material parallel to the septal surface with the aid of a binocular microscope and interpreted oxygen isotope variability in its shell to record daily depth migration behavior, but did not relate oxygen isotope variability to banding within the septa. Therefore, the data were unconstrained in time. The external spiral could not be sampled in such a manner to resolve depth migration because growth bands are inclined relative to the outer surface (Fig 1).

Mollusk shells are composed of two major layers: the outer prismatic layer (also known as the spherulitic prismatic layer [23]), positioned away from the chamber containing the body of the mollusk, and the inner nacreous layer that lines the body chamber [24] (Fig 1). The inner nacreous layer is composed of thin (<1 μm) interlocking tablets of aragonite that are surrounded by an organic framework. In *Nautilus*, the nacreous layer grows at a very shallow angle relative to the outer shell surface causing growth banding within it to be very thin [25] (Fig 1). The outer prismatic layer is composed of larger interlocking crystals of aragonite that has growth banding that forms at a higher angle relative to the outer shell wall, causing banding to be thicker and therefore permitting SIMS sampling within bands [25] (Fig 1). Growth of both the prismatic and nacreous layers in *Nautilus* shell occurs in the space enclosed by the mantle and periostracum at the apertural margin [23].

Growth banding with a variety of periods is widespread in biogenic carbonates. Circadian rhythms appear deep in the tree of life [26] and modulate metabolic processes related to growth [27]. Daily growth banding and variation of growth band width have previously been reported from several other mollusks. Intraprismatic banding in gastropod mollusks is daily and isochronous across the prismatic layer [28–30]. Statolith growth banding is daily in *Loliginidae* [31] and widths co-vary with food abundance [32]. Growth banding in bivalves has been widely studied [30,33–37]. Bivalves have daily growth banding and widths are modulated by seasonal temperature change, age, food availability and tides [29,30,38]. Growth banding in externally shelled cephalopod mollusks has only received minor attention and the periodicity and width of growth banding within the prismatic layer has not been reported for *Nautilus* [23,39–42]. External growth bands (i.e. those visible on the outer surface of the shell) in *Nautilus* are added every two days and have widths of twice that of measured daily growth rates [25,42]. External growth banding is visible in patterns of surface topography, while internal growth banding is visible due to changes in the ratio of organic matter to carbonate in the shell [22,23,43]. Growth banding visible within the outer prismatic layer of *Nautilus* shell is likely to form daily because of similarity in structure to daily growth bands in other mollusks and circadian rhythms are widespread in the tree of life.

This study investigates high spatial (10-μm scale) and high temporal resolution (sub-daily) oxygen isotope variation in *Nautilus* shell. We relate *in situ* δ^18^O variability to daily growth banding imaged in the outer prismatic layer of *Nautilus* shell. We sampled one wild-caught *Nautilus macromphalus* from New Caledonia and one aquarium-reared *Nautilus belauensis* [12,44] using a secondary ion mass spectrometer (SIMS) to measure δ^18^O in sampling spots with high precision (~0.3‰ 2SD). The high spatial resolution allows us to test for the record of depth-migration behavior in the shell of a mobile modern cephalopod and creates a framework for determining behavior through the analysis of stable isotopes in the extinct ammonites and historically collected *Nautilus*. This work will allow the interpretation of behavior and water column utilization independently from morphological inference, and may allow us to interpret near-surface temperature stratification.

## Materials and Methods

### Ethics statement

The specimen of *Nautilus macromphalus* (Mollusca: Cephalopoda) used in this study was live captured in a baited trap by Dr. Royal H. Mapes in 2002 before the species was given locally protected status around New Caledonia in 2008. The individual was euthanized and kept for a morphological study of *Nautilus macromphalus*. The sample has been deposited at the American Museum of Natural History (AMNH 105621). The SIMS mount from this sample is housed in the University of Wisconsin Geology museum with the number UW 2009 / 1.2. The *Nautilus belauensis* was hatched from an egg laid in captivity at the Waikiki aquarium as part of the first group of *Nautilus* hatched in captivity in 1990 [45]; however, the individual died for unknown reasons within a month of hatching. The sample is now housed at the American Museum of Natural History (AMNH 102555).

### Sample Material

**Fig 2:**
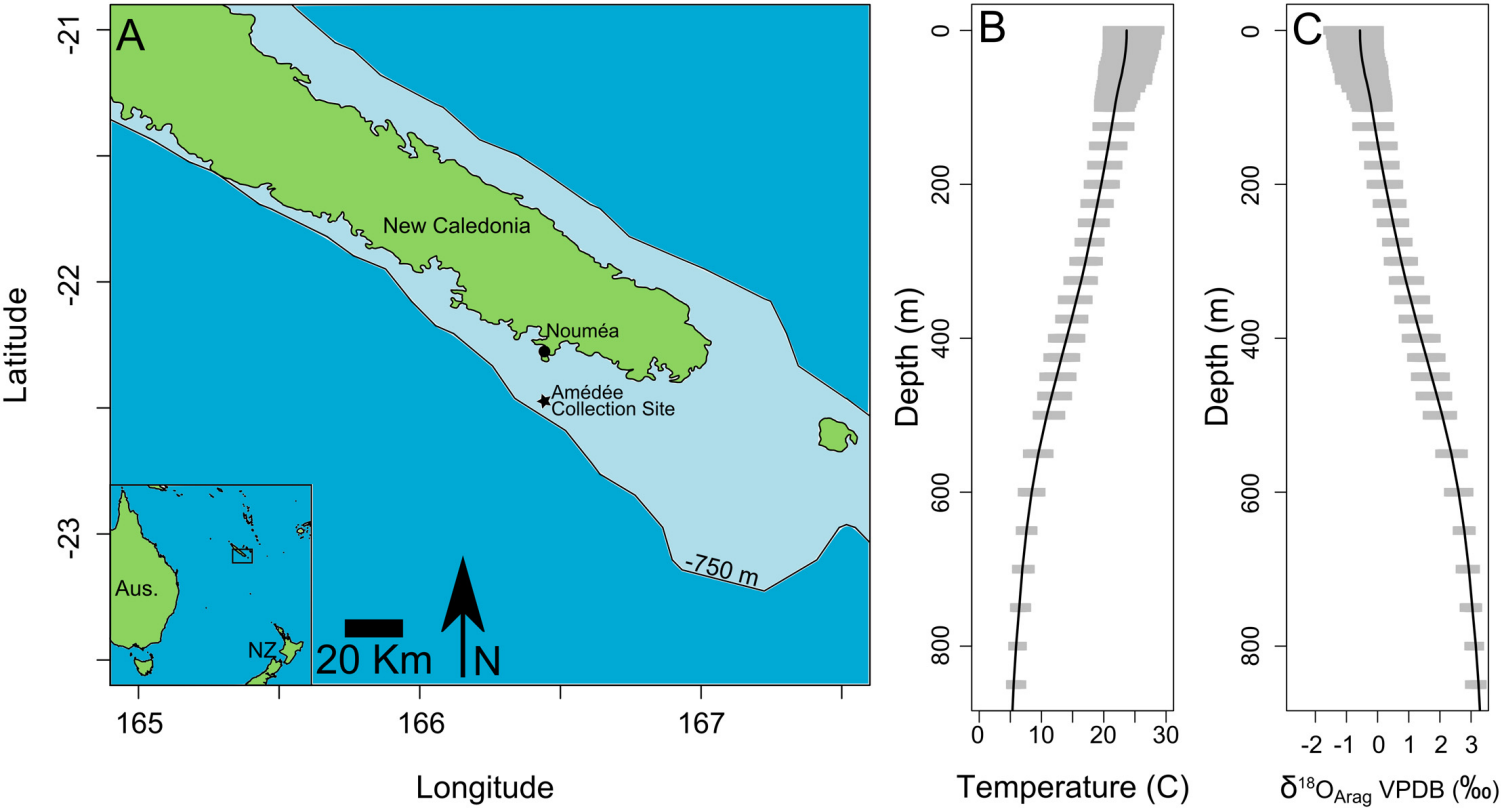
Map of the collection location of *Nautilus macromphalus* (AMNH 105621) near New Caledonia with δ^18^O_Arg_ predicted for the temperatures observed in the water depths *Nautilus* inhabits. A) Map of the southern end of New Caledonia showing the collection site (Amédée) of the *Nautilus macromphalus* used in this study. The 750 m bathymetry is the approximate lower limit of *Nautilus* habitat in the area defined by implosion depth. B) Water temperature for the map area [15] varies between the surface to 840 m depth. Gray bars indicate the approximate seasonal range in temperatures in the map area at each depth. The black line shows the average annual temperature with depth. A higher amount of seasonal variability is present in the upper 100 meters. C) The expected oxygen isotope ratio calculated using Eq 1 for an aragonite precipitated in isotope equilibrium with seawater at the temperatures shown in B assuming a constant δ^18^O_SW_ (0.5‰ VSMOW) with depth. The δ^18^O_Arg_ will vary by up to 4‰ over the range of 20°C for the depths that *Nautilus* can inhabit. The gray bars show expected seasonal variability in δ^18^O_Arg_ and the black line is the average expected δ^18^O_Arg_ with depth in the map area.

The wild-caught *Nautilus macromphalus* (AMNH 105621) was live captured in July, 2002 ~20 km south of Nouméa, New Caledonia (Amédée Lighthouse, New Caledonia 22°29’S, 166°27’E, Fig 2). The viscera were removed and the shell was stored dry (Royal H. Mapes pers. comm.). Water temperature near New Caledonia varies with depth (Fig 2) [15]. Temperatures at and near the surface (< 50 m), exhibit a seasonal range. The annual range in surface temperatures spans from 26.3°C to 22.5°C. Temperatures deeper in the water column (<50 m) exhibit minimal (<1°C) temperature seasonality (Fig 2) [15]. The difference between average annual surface water temperature and average annual temperature at 800 m depth is 17.5°C. Surface water δ^18^O values (δ^18^O_SW_) of 0.53 ±0.06 ‰ VSMOW (n = 4 from September to December 1999) were previously reported near the collection site [46] (Fig 2). There are no published seasonal data for δ^18^O_SW_ with depth off the coast of New Caledonia, but a seasonal range of 0.16‰ has been predicted from salinity [46]. These data suggest that within the wild-caught *Nautilus macromphalus*, δ^18^O_Arg_ variation should record vertical movement during depth migration behavior.

The *Nautilus belauensis* (AMNH 102555) was grown in an 80-liter all-glass aquarium. The aquarium temperature (22°C ±1°C) and one oxygen isotope analysis of the aquarium water (-0.2‰ VSMOW) were reported by Landman et al. [12]. About once a week the water lost due to evaporation (~10%) and remaining water were replaced over an 8-hour period [45,47]. These data about aquarium conditions suggest that variation of δ^18^O_Arg_ within the aquarium-reared sample could be the result of changes in δ^18^O_water_ due to evaporation from the tank.

The study of the stable isotope geochemistry of *Nautilus* has a long history [5]. Evidence for oxygen isotope equilibrium precipitation of aragonite has been observed for both wild [1–4,6,11,48,49] and for aquarium-reared specimens [12]. Although none of the studies to date have grown *Nautilus* in water with continuous measurement of both δ^18^O and temperature, it is reasonable to assume that aragonite is precipitated in, or close to, oxygen isotope equilibrium with seawater at ambient temperature given similarity in measurements from field and aquarium data [1,4–8,12]. The calibration of Kim et al. [50] will be used for the equilibrium fractionation of aragonite and water because it was calibrated over a broad temperature range (0–40°C). This calibration also falls within statistical error of the commonly-used formula derived by Grossman and Ku [51]. The Kim et al. [50] equation is as follows:

1000lna(O18)aragonite−water = 17.88 ± 0.13 (103T) – 31.14  ±  0.46 (1)

where temperature (T) is in K. Aragonite δ^18^O values are reported vs. VPDB. Seawater δ^18^O is reported vs. VSMOW. The δ^18^O values of seawater and shell aragonite must be reported relative to the same standard to estimate temperature using Eq 1. The conversion between VPDB and VSMOW after Coplen [52], is:

$$\delta^{\text{18}}O_{VSMOW}=\text{1.03091 }*\;\delta^{\text{18}}O_{VPDB}\;+\text{ 30.91}$$ (2)

where δ^18^O_VSMOW_ is the oxygen isotope value reported relative to the Vienna Standard Mean Ocean Water (VSMOW) and δ^18^O_VPDB_ is the oxygen isotope value reported relative to the Vienna PeeDee Belemnite (VPDB).

### Preparation of SIMS mounts

SIMS sample mounts of both shells were created from chips removed from sections near the medial plane and near the aperture to maximize the width of the growth bands and growth rate [53]. The analyzed section from the wild-caught *Nautilus macromphalus* was sampled ~3.5 cm from the growth edge, and represents growth in ~December 2001, assuming constant ~182 μm/day growth. The section from aquarium-reared *Nautilus belauensis* was removed from post-hatching growth. Both shells were roasted separately at 320°C for one hour under vacuum to remove volatile organic matter (after [54,55]). The roasted samples were cast in the center of two, 2.5 cm-diameter epoxy mounts with two grains of UWC-3 calcite standard (δ^18^O = 12.49 ‰, VSMOW; [56]). The surfaces to be analyzed were placed within 5 mm of the center of the mount to minimize instrumental bias of SIMS data caused by sample position [57]. Sample relief was minimized (≤ 1 μm) to avoid topographic effects on measured δ^18^O [57]. Initial sectioning of the wild-caught sample through the medial plane (Fig 1) was done with a bandsaw. Growth banding was difficult to image in confocal laser fluorescent microscopy (CLFM) from surfaces cut by the bandsaw even after epoxy mounting and polishing. The saw removed ~2 mm of the center of the shell and damaged adjacent surfaces making banding difficult to image. Polishing of these surfaces removed less than 50 μm of material. The best sample surfaces for imaging and SIMS analysis did not come from the initial surface cut by the bandsaw, but were produced from shell material that was completely encased in epoxy before being cut with a Buehler Isomet^®^ low speed, water cooled saw and that was subsequently polished and finished on a vibrating polisher with a colloidal alumina polish (0.05 μm).

### Sample Imaging

**Fig 3:**
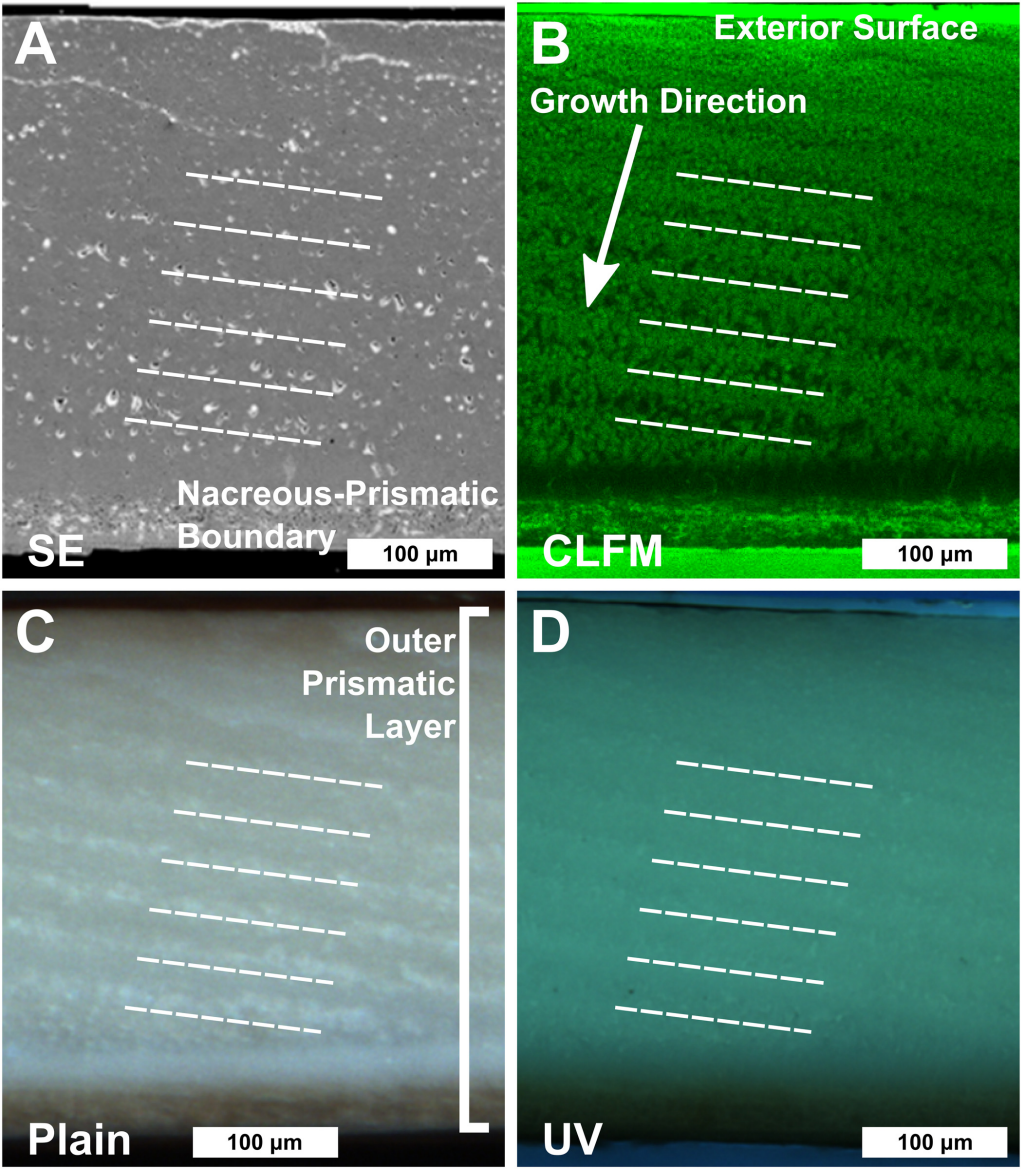
The outer prismatic layer of a portion of the polished surface from *Nautilus macromphalus* (AMNH 105621) imaged by four different techniques: A) SE, B) CLFM, C) Plain light dissecting microscope, D) UV. The oldest shell precipitated is in the upper right corner of all images. Growth proceeds to the lower left. All images are of the same portion of the shell. Five bands are highlighted with white lines. A) SE image showing the polished shell surface. Lines of pores in the SE image (A) correlate to low fluorescence in CLFM (B), bright portions of the band in the plain light correlate to dissecting microscope image (C) and dark portions in the long wavelength UV (~360 nm) fluorescence image (D). These cavities are the same as those intersected by some SIMS analysis pits (Fig 4). B) CLFM (true color) image showing growth bands. Growth bands extend from the exterior surface of the outer prismatic layer (top of the image) to the interior boundary with the nacreous layer. Note that the banding closer to the nacreous boundary is more pronounced than the banding toward the exterior of the shell. C) Plain-light dissecting microscope image (true color) of polished shell surface. Banding is apparent but discontinuous. D) UV fluorescence (true color) image of the shell. Faint banding is present with an orientation equal to that found in other imaging techniques. CLFM images on the uncoated sample mount were made using a Bio-Rad MRC-1024 scanning confocal microscope at the W. M. Keck Laboratory for Biological Imaging at UW-Madison operated with a 40 mW laser at a wavelength of 488 nm. Images of banding within the outer prismatic layer were best expressed through an emission filter that detects visible green light (λ = 505 to 539 nm). Growth-band orientation in the prismatic layer was used to place SIMS transects.

The polished *Nautilus* shell surfaces were investigated by several techniques to preselect locations for SIMS analysis pits relative to growth banding, determine carbonate mineralogy (calcite vs. aragonite), and to avoid sampling on surface features such as cavities or cracks [58]. Initial analysis was done by scanning electron microscopy (SEM) at UW-Madison, Dept. of Geoscience, utilizing secondary electron (SE) (Fig 3A), cathodoluminescence (CL), backscattered electron (BSE), and electron backscatter diffraction (EBSD) detectors. SE images were used to avoid cracks and cavities. EBSD indicated that the shell material was aragonite and had not changed to calcite during roasting [59]. Growth banding was not clearly resolved by SEM and was imaged in more detail by three other techniques: (1) Confocal laser fluorescent microscopy (CLFM) (Fig 3B), (2) long wavelength (~350 nm) Ultraviolet microscopy (UV) (Fig 3C), and (3) a dissecting microscope (Fig 3D).

### Image Analysis

Images in this study were important in two ways: 1) to guide the placement of SIMS analysis locations by showing the orientation of growth banding and locations of cavities and 2) to provide information on the location of SIMS pits relative to growth banding, which can serve as a chronometer. Images for the wild-caught *Nautilus macromphalus* and aquarium-reared *Nautilus belauensis* are shown in S1 and S2 Figs, respectively. SEM images were specifically used to determine the best placement of SIMS analysis pits to avoid cavities, and CLFM images were used to guide the placement relative to growth banding in order to sample as much growth as possible with sufficient density. ImageJ (version 1.43u [60]) was used to measure the intensity of fluorescence from the monochrome green-black CLFM-image, with values ranging from 255 (Bright green) to 0 (black). Lines 1000 pixels (9 pixels/μm) long and one pixel wide parallel to growth banding were averaged by ImageJ to minimize the influence of local variation in luminescence (Fig 1A and 1B). A unified linear reference frame (i.e. distance along a straight line) perpendicular to banding was created by correlating images along growth bands. Multiple CLFM images were required to construct the composite record (S2 Fig). Image-to-image and across-image variation in luminosity was removed by subtracting a lowess regression (to smooth data) through the dataset, leaving the residual variation in luminosity from individual growth bands [61,62]. CLFM luminosity values were matched to the center of SIMS δ^18^O_Arg_ pits by equal distance along the axis perpendicular to growth banding.

### SIMS analysis of oxygen isotope ratios

Oxygen isotope ratios were analyzed by SIMS at the WiscSIMS Laboratory, Dept. of Geoscience, UW-Madison, using a CAMECA ims-1280 large-radius multicollector secondary ion mass spectrometer (SIMS). Instrumental conditions were similar to those described by Orland et al. [63] and Kita et al. [57]. A ~1.3 nA primary beam of ^133^Cs^+^ was focused to spots of ~10 μm diameter on the sample surface. Two Faraday Cup detectors were used to simultaneously collect ^18^O^-^ and ^16^O^-^ions during all sessions. In 2013 and 2014, one additional Faraday Cup was used to collect ^16^O^1^H^-^ ions. Pits were sputtered to a depth of ~1 μm. Surface charging was compensated by an electron flood gun in combination with a ~20 nm thick carbon coat (*Nautilus macromphalus* in 2011) or gold coat (*Nautilus macromphalus* in 2014, *Nautilus belauensis* in 2013) which was applied after final polishing and surficial cleaning. Surficial cleaning with deionized water and ethanol was brief to minimize risk of surface etching.

A total of 571 SIMS analyses of δ^18^O were made: 193 on standards, 295 analyses on the wild-caught sample and 84 analyses on the aquarium-reared sample. Analyses on the wild-caught sample were performed over two sessions, 6/28/2011-6/29/2011 and 2/14/2014-2/15/2014. Analyses on the aquarium-reared sample were conducted on 9/25/2013 and 9/26/2013. Groups of 10–15 sample analyses were bracketed by four analyses before and four analyses after of UWC-3 calcite standard. The precision of the eight bracketing standard analyses was used to define the spot-to-spot precision of sample analyses [64] that averaged ±0.3‰ (±2 SD) for this dataset. The UWC-3 calcite standard [56] was used as a running standard for aragonite. A difference in instrumental bias (sometimes called Instrumental Mass Fractionation) between the two polymorphs has been observed [59,65]. The UW Arg-7 aragonite standard was compared to the UWC-3 calcite standard (δ^18^O = 12.49 ‰, VSMOW; [56]) on 9/25/2013 when the aquarium-reared sample was analyzed and the wild-caught sample was analyzed on 2/14/2014. We recalibrated the UW Arg-7 standard to δ^18^O = 19.73 ‰ (VSMOW) from δ^18^O = 20.03 ‰ (VSMOW) as reported by Orland [65]. Orland [65] calibrated this standard from analyses at the University of Wisconsin-Madison using the phosphoric acid fractionation factor for aragonite at 25°C (α_CO2(Acid-Aragonite)_ = 1.01034) reported by Freidman and O’Neil [66]. Here we use the phosphoric acid fractionation factor from Kim et al. [67] at 25°C (α_CO2(Acid-Aragonite)_ = 1.01063) to recalibrate the value because Kim et al. [67] dataset was large (n = 29 for aragonite) and therefore reduced statistical uncertainty by a factor of 3.8 over the value reported by Freidman and O’Neil [66]. The difference in SIMS instrumental bias between aragonite and calcite was 0.61‰ for the session when the aquarium reared sample was analyzed, and 0.57‰ when the wild-caught sample was analyzed. The difference in bias between calcite and aragonite (0.81‰) for data collected in 2011 [68] was estimated by comparing the mean δ^18^O_Arg_ values of populations (6/28/2011-6/29/2011 vs. 2/14/2014-2/15/2014) of very closely spaced analyses on the shell.

### Evaluation of the SIMS analysis pits in *Nautilus* shell

**Fig 4:**
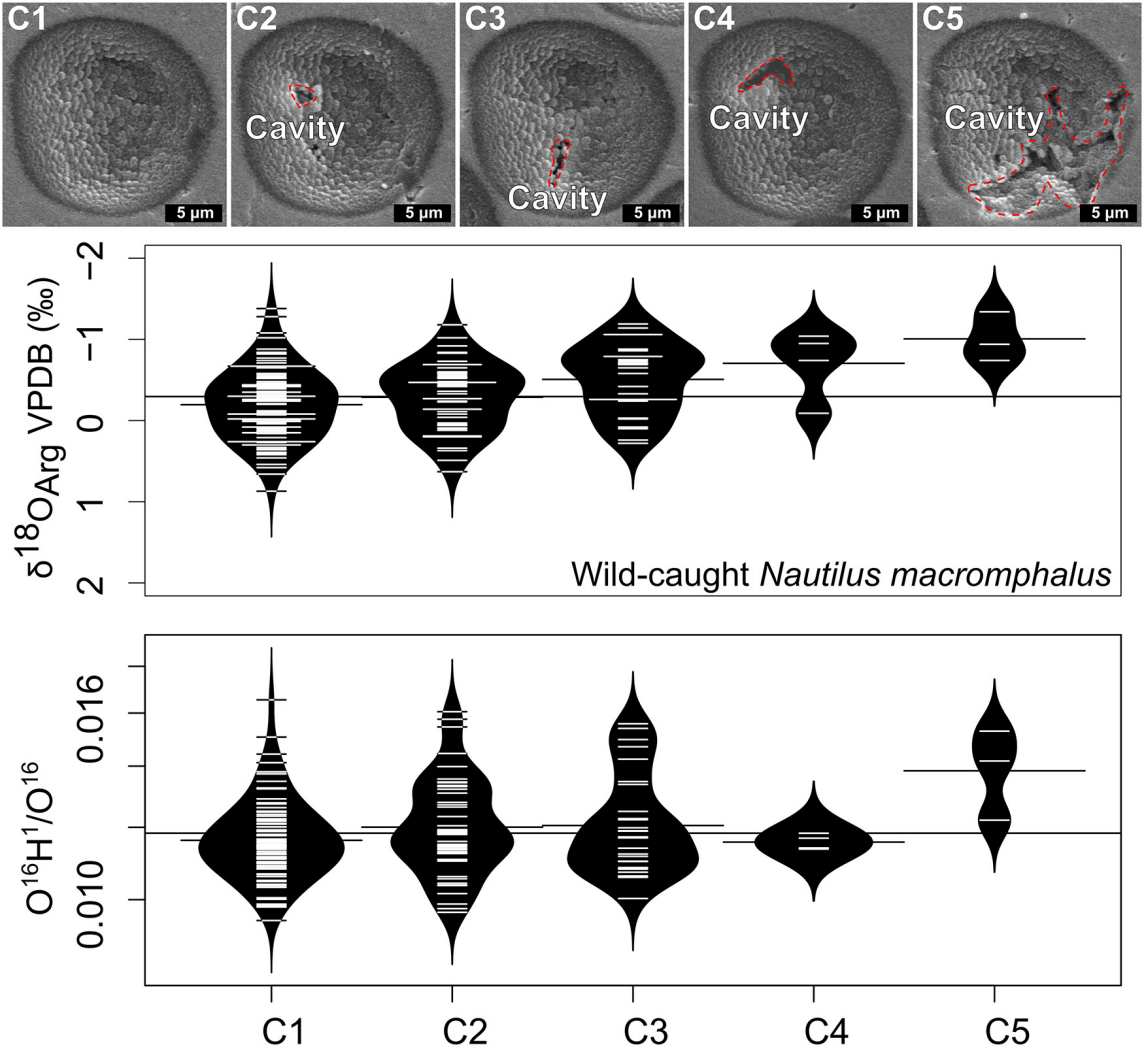
Representative SE images illustrating the criteria used for qualitative SIMS pit categorization for both the wild-caught and aquarium-reared *Nautilus* and beanplots showing the distribution of δ^18^O_Arg_ and O^16^H^1^/O^16^ across pit categories from *Nautilus macromphalus* (AMNH 105621). Cavities that the SIMS analysis pits intersected are likely left from the removal of organic matter during roasting or from plucking during polishing. Organic matter in the outer prismatic layer of mollusks has banding but also irregular clumps [22]. In the images above, cavities are outlined by a red dashed line. All pits are approximately 1-μm deep. Category 1 (C1) This pit is classified as regular in appearance because it has a uniform texture throughout the walls and bottom of the pit (wild-caught, *n = 134*; aquarium-reared, *n = 25*). Category 2 (C2) This pit minimally intersects two small cavities and therefore is also classified as regular in appearance (wild-caught, *n = 85*; aquarium-reared, *n = 5*). Category 3 (C3) A more significant intersection with a cavity at the bottom of this analysis pit makes it irregular (wild-caught, *n = 52*; aquarium-reared, *n = 16*). Category 4 (C4) A larger intersection with a cavity, but this cavity intersection is on the wall of the pit rather than on the bottom. This pit is also considered to be irregular and 5% of analyses had cavity intersections similar to this pit (wild-caught, *n = 14*; aquarium-reared, *n = 7*). Category 5 (C5) This pit is an example of some of the most extreme pit-cavity intersections and is a highly irregular pit (wild-caught, *n = 8*; aquarium-reared, *n = 0*). Beanplots [71] below show the estimated density distribution of δ^18^O_Arg_ (top) or O^16^H^1^/O^16^ (bottom) for the wild-caught *Nautilus macromphalus* where we have paired analyses (2/14/2014-2/15/2014). The thin black line extending across the beanplots indicates the mean for all analyses, short lines extending from each category indicate each category mean, and short white lines within each category indicate individual observations.

SIMS analysis pits were imaged by SEM to screen for irregular pit shapes before interpretation of the data (Fig 4, S1 and S2 Figs) [58]. Data from SIMS pits that intersect cavities, cracks, or inclusions are not guaranteed to be reliable [56,69,70]. Cracks were readily avoided during analysis; however, the roasting procedure appears to have removed intercrystalline organic matter, leaving small cavities that were difficult to avoid. Five pits are displayed that are representative examples for the range of pit-cavity interaction and these were used as a guide for the classification of the remaining pits (Fig 4). Pit categories were based on inspection of SEM images only and not on measured δ^18^O_Arg_. Pits designated category 1 (C1) have no cavity intersection visible by SEM. Pits designated category 2 (C2) intersect small cavities (< 1 μm) on the edge of the pit. Category 3 (C3) pits intersect slightly larger cavities (~1 μm) in the bottom or sides of the pit. Pits in category 4 (C4) intersect cavities that are 2–3 μm wide. Pits designated category 5 (C5) intersect cavities in the shell larger that are than 3 μm. An additional filter of >95% yield relative to the bracketing UWC-3 calcite data was also applied to data from both the wild-caught and aquarium-reared samples to screen for irreproducible δ^18^O_Arg_ measurements [58].

## Results

### Growth banding

Banding within the outer prismatic layer is apparent in CLFM, plain light on a binocular microscope, and UV light fluorescence microscopy (Fig 3). The banding intersects the exterior surface of the shell and the nacreous-prismatic boundary at low angles (~11°). A single band is defined by the change in fluorescence from low to high and back to low again (Figs 1 and 3B). The number and distribution of bands observed in the outer prismatic layer is equal for all of the imaging techniques, but clarity of banding differs between imaging techniques. Banding is not readily discernible in SEM (Fig 3A), but there are concentrations of cavities that have the same orientation and spacing as banding visible under other imaging techniques. Cavities are defined as holes in the shell carbonate visible in SE images (black spots with white rims, from charging in Fig 3A). Bands of cavities correlate with low fluorescence in CLFM, the dark portion of bands in UV and the bright portion of the bands in plain light. CLFM produced images of banding that are the clearest out of any technique (Fig 3B). The banding is also apparent in plain light, but some bands are not very pronounced (Fig 3C). Banding is also discernible in long-wavelength (~360 nm) UV (Fig 3D), but the delineation of band edges was less pronounced in unadjusted (contrast—brightness unenhanced) images than in plain light. The visibility of banding dramatically improved over bandsaw cut edges when shell was cut by a Buehler Isomet^®^ low speed diamond saw and subsequently polished and finished with a colloidal alumina polish (0.05 μm).

The intraprismatic growth banding of the wild-caught sample of *Nautilus macromphalus* is readily visible in CLFM images. The contrast between low fluorescence and high fluorescence portion of each band varies between the end of the band near the prismatic-nacreous boundary (Fig 1, Box B) and the exterior surface of the shell (Fig 1, Box A). The contrast between low fluorescence and high fluorescence portions of bands is greater near the prismatic-nacreous boundary and lower toward the exterior shell surface. The apparent ‘graininess’ of bands, i.e. the size of shapes with uniform or nearly uniform fluorescence, also varies along the expression of a single band. Graininess is low toward the exterior of the shell and increases toward the prismatic-nacreous boundary.

#### Apertural growth rate estimated by CLFM Imaging

The thickness of well-expressed bands in CLFM averages 34.7 μm (measured normal to bands) across the sampled portion of the wild-caught *Nautilus macromphalus* with a standard deviation of 8.6 μm and a range from 19.8 to 58.9 μm. Growth band thickness can be compared to published apertural growth rates if width is converted to distance in the apertural direction. An average apertural growth of 182 μm per band is obtained using the angle of banding intersection (~11°) and band width (34.7 μm) (Fig 1). Apertural growth per band in the wild-caught *Nautilus macromphalus* is between 104 and 308 μm. In the aquarium-reared sample, growth banding in CLFM averages 20.1 μm (measured normal to banding) across 14 bands with a standard deviation of 6.1 μm and a range of 12.4 to 30.7 μm. This translates to a growth rate of 105 μm/day and a range from 65 to 160 μm/day assuming banding is daily. Growth rates reported in literature span from 80 to 300 μm per day and nearly encompass the entire range of values calculated from growth band width in these two individuals [21,42,72–74].

### Oxygen Isotope Ratios

#### Evaluation of SIMS pit appearance as criteria for acceptance or rejection of data

Analysis pit locations were selected carefully using the reflected light optics of the IMS-1280 during analysis and correlating with images made of porosity before analysis. However, 2-μm-scale features are difficult to see [75] in the coated sample surface and 74 (25%) of the 295 analyses on the wild-caught sample intersected cavities that were identified by SEM after analysis. Perhaps due to higher cavity density or more evenly distributed small cavities, 23 (43%) of 53 analyses on the aquarium-reared sample intersected subsurface cavities. Five categories were qualitatively defined by the amount of pit intersection and pits were assigned to a category by inspection of SEM images (Fig 4).

A systematic trend of decreasing δ^18^O_Arg_ is present with increasing cavity intersection (Fig 4) (ANOVA wild-caught p<0.001, aquarium-reared, p = 0.014). Mean δ^18^O_Arg_ for the five categories, listed in increasing amount of intersection with cavities for the wild-caught sample, are: -0.3‰ (C1, n = 134); -0.5‰ (C2, n = 85); -0.6‰ (C3, n = 52); -1.1‰ (C4, n = 14); and -2.3‰ (C5, n = 8). Mean δ^18^O_Arg_ for the aquarium-reared sample pit categories, are -1.3‰ (C1, n = 25), -1.5‰ (C2, n = 5), -1.4 (C3, n = 16), -1.9 (C4, n = 7), and no points were classified as C5. In the wild-caught *Nautilus macromphalus*, the mean δ^18^O_Arg_ of pits with the most cavity intersection (C5) is 2‰ lower than that of the group with the least cavity intersection (C1). In the aquarium-reared *Nautilus belauensis*, the mean δ^18^O_Arg_ of pits with the most cavity intersection (C4) is ~0.5‰ lower than that of the group with the least cavity intersection (C1). Analyses from irregular pits (C3, C4, C5) may not accurately measure the δ^18^O of aragonite. Although cavities appear to correlate to areas with lower CLFM luminosity (Fig 3), irregular pits are not grouped in areas with high or low CLFM brightness (ANOVA wild-caught p = 0.12; aquarium-reared p = 0.13). The two categories of pits with minimal cavity intersection (C1 and C2, aquarium-reared sample n = 30, Table 1, wild-caught sample n = 219, Table 2) are considered regular and will be discussed further. Exclusion of pits in categories C3, C4 and C5 (Fig 3) does not significantly alter the interpretation of this dataset for either wild-caught or aquarium reared specimens and inclusion of these would only increase the variation observed in δ^18^O_Arg_, especially within the wild-caught specimen. The complete dataset for the wild-caught *Nautilus macromphalus* (AMNH 105621) is in S1 Table and the complete dataset for the aquarium-reared *Nautilus belauensis* (AMNH 102555) is in S2 Table.

**Table 1:** Summary of SIMS analyses of oxygen isotope ratios measured at WiscSIMS on the *Nautilus belauensis* (AMNH 102555) and confocal microscope brightness. These are ion microprobe analyses of oxygen isotope ratios, grey scale values from confocal laser fluorescence images, and distance perpendicular to banding for analyses that were shown to be regular based on pit morphology. See supplementary material for a complete list of measurements.

Analysis Name	δ^18^O_VPDB_** (‰)	δ^18^O_VSMOW_* (‰)	Error^£^ (2s.d.)	δ^18^O_Raw_^λ^ (‰)	Relative Yield^€^ (%)	Pit category^Ω^	Dist. ^₭^ (μm)	CLFM Greyscale^
2–9	-1.7	29.2	0.3	27.1	97.60	1	8.1	0.40
2–6	-1.0	29.9	0.3	27.8	95.00	1	49.4	2.81
2–5	-1.7	29.2	0.3	27.2	95.70	2	54.2	-0.81
3–12	-1.4	29.5	0.4	27.4	96.60	1	60.5	-4.19
3–10	-0.8	30.1	0.4	28.0	96.30	1	65.3	-4.52
2–4	-0.8	30.1	0.3	28.1	95.30	1	66.1	-4.31
3–9	-0.8	30.1	0.4	28.0	95.80	1	69.2	-0.20
3–8	-0.7	30.2	0.4	28.1	96.10	1	78.8	9.39
3–7	-1.0	29.9	0.4	27.8	95.50	2	87.5	-6.69
3–6	-0.9	30.0	0.3	27.9	96.70	1	99.4	0.98
3–5	-0.8	30.1	0.3	28.0	95.60	1	107.3	-2.84
3–13	-1.6	29.3	0.4	27.2	96.20	1	108.1	-1.81
1–10	-1.1	29.8	0.4	27.8	96.60	1	116.9	1.12
1–9	-1.6	29.3	0.4	27.3	97.80	1	125.6	-3.90
3–3	-1.4	29.5	0.3	27.4	96.20	1	132.7	1.13
1–8	-1.4	29.5	0.4	27.5	97.80	1	134.3	0.75
3–2	-1.5	29.3	0.3	27.2	95.70	1	139.9	2.48
1–7	-1.5	29.4	0.4	27.4	97.50	1	143.0	8.08
3–1	-1.8	29.1	0.3	27.0	95.90	1	148.6	-7.16
1–4	-1.8	29.1	0.4	27.1	96.90	1	174.0	0.84
1–3	-1.8	29.1	0.4	27.1	97.30	2	189.9	-1.39
4–14	-1.9	29.0	0.2	27.1	98.30	1	208.9	-3.36
4–8	-1.9	29.0	0.3	27.1	98.10	1	216.9	0.66
4–18	-1.1	29.8	0.2	27.9	95.40	1	228.8	1.66
4–7	-2.2	28.7	0.3	26.8	98.70	1	232.7	0.06
4–12	-1.6	29.3	0.2	27.4	97.00	2	236.7	-2.80
4–5	-1.5	29.4	0.3	27.5	97.60	2	258.9	-6.19
4–3	-1.4	29.5	0.3	27.6	97.80	1	280.3	-0.98

Analysis names refer to Transect#-Pit#.

** Conversion from VSMOW and corrected to aragonite δ^18^O_VPDB_ = (δ^18^O_VSMOW_− 30.91)/1.03091.

* Value adjusted by bracketing UWC-3 calcite standard and corrected to aragonite (-0.61‰), see text.

^λ^ Value reported vs. VSMOW δ^18^O_raw_ = {(^18^/_16_ measured/0.00200520)-1}*1000

^£^ Precision for δ^18^O_VPDB_ measurements derived from 2 times standard deviation of eight bracketing standards.

^€^ Yield (counts per second/nA, a measure of ionization efficiency) relative to the mean yield of 8 bracketing standards for UWC-3 calcite.

^Ω^ Pit category defined by SEM images compared to images in Fig 4.

^₭^ Distance values in micrometers perpendicular to visible banding to center of analysis pit.

^ Normalized CLFM grey scale value from averaged line of pixels parallel to growth banding in CLFM image for pit location with lowess regression removed to correct for trends in brightness across and between images. Low values are dark portions of growth bands and high values are bright portion.

**Table 2:** Summary of SIMS analyses of oxygen isotope ratios measured at WiscSIMS on the *Nautilus macromphalus* (AMNH 105621) and confocal microscope brightness. These are ion microprobe analyses of oxygen isotope ratios, grey scale values from confocal laser fluorescence images, and distance perpendicular to banding for analyses that were shown to be regular based on pit morphology. See supplementary material for a complete list of measurements.

Analysis Name	δ^18^O_VPDB_** (‰)	δ^18^O_VSMOW_* (‰)	Error^£^ (2s.d.)	δ^18^O_Raw_ ^λ^ (‰)	Date	RelativeYield^€^ (%)	Dist.^₭^ (μm)	Pit quality	CLFM Greyscale^
0–1	-0.7	30.2	0.2	26.9	2/14/2014	97.00	23.7	1	1.61
0–2	0.0	31.0	0.2	27.7	2/14/2014	97.00	36.2	1	1.43
0–3	-0.4	30.5	0.2	27.2	2/14/2014	96.70	38.0	1	3.11
1–1	-0.8	30.1	0.2	25.3	6/28/2011	95.36	38.0	1	3.11
1–2	-1.3	29.6	0.2	24.8	6/28/2011	95.31	43.8	2	3.41
0–4	0.3	31.2	0.2	27.9	2/14/2014	96.10	47.9	1	2.62
0–5	0.2	31.1	0.2	27.8	2/14/2014	96.70	77.5	1	3.96
1.5–1	-0.1	30.8	0.5	27.8	2/14/2014	95.30	80.2	1	2.73
1–7	-1.0	29.9	0.2	25.1	6/28/2011	95.29	87.8	1	-3.15
0–6	0.1	31.0	0.2	27.7	2/14/2014	96.70	91.4	2	-4.18
1.5–2	-0.7	30.1	0.5	27.1	2/14/2014	96.40	96.7	1	-2.58
0–7	0.4	31.3	0.2	28.0	2/14/2014	97.00	106.6	1	4.04
0–8	0.0	30.9	0.2	27.7	2/14/2014	96.70	108.9	1	4.29
1.5–3	-0.3	30.6	0.5	27.6	2/14/2014	96.10	112.0	1	2.00
0–9	0.1	31.0	0.2	27.7	2/14/2014	97.60	126.8	1	-1.95
1–11	-1.3	29.6	0.2	24.7	6/28/2011	95.52	126.8	2	-1.95
1.5–5	0.1	31.0	0.5	28.0	2/14/2014	97.90	130.4	2	-0.92
0–10	0.2	31.1	0.2	27.8	2/14/2014	96.90	141.6	2	3.30
0–11	-0.2	30.7	0.5	27.6	2/14/2014	97.10	153.8	2	-1.80
2–15	-0.7	30.2	0.1	25.4	6/29/2011	96.17	166.3	1	-1.55
1–15	-1.4	29.4	0.2	24.6	6/28/2011	95.46	171.2	2	0.64
0–13	-0.7	30.2	0.5	27.2	2/14/2014	97.50	172.5	2	1.78
1.5–7	-0.1	30.8	0.5	27.7	2/14/2014	96.60	173.0	2	1.74
2–3	-0.7	30.2	0.2	25.3	6/28/2011	95.36	177.5	2	0.20
2–2	-0.6	30.2	0.2	25.4	6/28/2011	96.14	179.3	2	0.28
1–16	-0.4	30.5	0.2	25.7	6/28/2011	95.47	179.7	2	0.24
0–14	0.3	31.3	0.5	28.2	2/14/2014	97.20	184.2	2	-0.70
0–15	-0.1	30.8	0.5	27.8	2/14/2014	97.40	187.3	2	0.22
2–17	-0.8	30.1	0.1	25.3	6/29/2011	96.37	189.1	2	-0.18
1.5–8	-0.6	30.3	0.5	27.3	2/14/2014	97.20	192.3	2	1.67
2–4	-1.1	29.8	0.2	25.0	6/28/2011	95.47	193.2	2	2.13
1.5–9	-0.8	30.1	0.5	27.0	2/14/2014	97.60	201.7	2	0.82
2–18	-0.9	30.0	0.1	25.1	6/29/2011	96.42	202.1	1	0.85
2–19	-0.9	30.0	0.1	25.2	6/29/2011	96.13	213.3	2	2.93
2–6	-0.2	30.7	0.3	25.8	6/28/2011	96.57	220.5	1	-3.48
0–18	-0.5	30.4	0.5	27.4	2/14/2014	97.20	222.3	2	-3.51
2–20	-0.7	30.2	0.1	25.4	6/29/2011	96.69	226.3	1	-4.81
2–21	0.0	30.9	0.1	26.1	6/29/2011	96.21	236.2	1	-0.56
0–20	-0.1	30.8	0.5	27.8	2/14/2014	98.60	238.9	2	-0.13
0–19	0.3	31.2	0.5	28.1	2/14/2014	97.20	242.5	1	2.25
2–8	-0.4	30.5	0.3	25.6	6/28/2011	96.69	245.2	1	1.92
2–9	-1.1	29.7	0.3	24.8	6/28/2011	95.73	257.3	1	0.05
2–23	-0.6	30.3	0.1	25.5	6/29/2011	96.98	260.0	2	-0.99
2–24	-1.2	29.7	0.1	24.9	6/29/2011	97.12	273.0	2	-0.96
2–13	-0.8	30.1	0.3	25.1	6/28/2011	95.99	285.1	1	-0.93
3–2	-0.3	30.6	0.2	25.6	6/29/2011	95.60	320.5	1	-1.91
3–13	-0.2	30.8	0.2	25.9	6/29/2011	96.09	335.8	1	2.91
3–4	-0.4	30.5	0.2	25.5	6/29/2011	96.17	344.7	1	5.99
3–14	-0.3	30.6	0.2	25.8	6/29/2011	96.60	346.5	2	4.45
3–6	-1.0	29.9	0.2	24.9	6/29/2011	96.83	366.3	1	-2.53
3–16	-0.8	30.1	0.2	25.2	6/29/2011	96.99	368.0	2	-0.30
3–7	-0.2	30.7	0.2	25.7	6/29/2011	96.37	377.9	1	2.72
3–8	0.0	30.9	0.2	25.9	6/29/2011	96.58	382.4	2	0.08
3–17	-0.4	30.5	0.2	25.6	6/29/2011	96.91	382.4	2	0.08
3–18	-0.6	30.3	0.2	25.4	6/29/2011	97.75	392.7	1	-2.86
3–19	-0.2	30.7	0.2	25.9	6/29/2011	97.16	400.3	1	1.16
3–9	-0.2	30.7	0.2	25.7	6/29/2011	95.74	404.8	1	2.48
3–20	-0.8	30.1	0.2	25.3	6/29/2011	96.57	419.2	1	-0.12
3–21	-0.9	30.0	0.2	25.2	6/29/2011	97.09	431.3	1	0.24
3–22	-0.5	30.4	0.2	25.5	6/29/2011	97.00	440.2	1	1.37
3–12	-0.5	30.3	0.2	25.4	6/29/2011	97.75	443.8	2	0.15
4–3	-0.5	30.4	0.2	25.6	6/29/2011	95.24	446.5	1	-0.90
3–23	-1.6	29.3	0.2	24.4	6/29/2011	95.23	449.2	2	-0.62
4–4	-1.2	29.7	0.2	24.9	6/29/2011	97.15	458.2	1	-3.71
3–24	-0.5	30.3	0.2	25.5	6/29/2011	98.01	465.4	1	-2.71
4–5	-0.6	30.3	0.2	25.6	6/29/2011	96.58	473.4	2	-0.11
4–6	-0.5	30.4	0.2	25.7	6/29/2011	96.40	482.8	2	-1.30
4–7	-0.5	30.3	0.2	25.6	6/29/2011	96.20	490.5	1	0.48
4–20	-0.6	30.3	0.2	25.5	6/29/2011	96.87	492.3	2	1.94
4–8	-0.5	30.4	0.2	25.6	6/29/2011	96.79	498.5	1	5.71
4–21	-0.7	30.2	0.2	25.4	6/29/2011	97.02	504.8	2	2.68
4–9	0.1	31.0	0.2	26.2	6/29/2011	96.76	512.4	1	-2.80
4–11	-0.5	30.4	0.2	25.6	6/29/2011	96.31	533.1	1	1.87
4–23	-0.3	30.6	0.2	25.8	6/29/2011	96.29	536.7	1	0.18
4–12	-0.5	30.4	0.2	25.7	6/29/2011	96.25	544.3	2	1.42
4–24	-0.4	30.5	0.2	25.7	6/29/2011	96.06	549.2	2	0.63
4–13	-0.7	30.2	0.1	25.5	6/29/2011	97.28	556.8	1	-1.79
4–14	-1.0	29.9	0.1	25.2	6/29/2011	96.58	564.5	1	-3.18
5–1	-1.2	29.7	0.1	25.0	6/29/2011	96.26	576.1	1	4.74
4–16	0.0	30.9	0.1	26.1	6/29/2011	96.71	577.5	1	4.52
4–26	-0.2	30.7	0.2	25.9	6/29/2011	97.08	585.5	1	0.50
5–4	-0.7	30.2	0.1	25.5	6/29/2011	97.40	594.8	2	-4.49
4–27	-0.5	30.4	0.2	25.6	6/29/2011	96.67	600.4	1	1.37
5–17	-0.5	30.4	0.2	25.6	6/29/2011	96.39	601.7	1	2.60
5–5	-0.5	30.4	0.1	25.7	6/29/2011	97.20	605.3	2	4.57
5–18	-0.4	30.5	0.2	25.7	6/29/2011	96.51	615.5	2	-0.33
5–6	-0.8	30.0	0.1	25.3	6/29/2011	98.62	621.2	1	-5.18
5–7	-0.7	30.2	0.1	25.5	6/29/2011	96.42	630.3	2	0.51
5–8	-0.8	30.0	0.1	25.3	6/29/2011	95.86	642.3	1	3.64
5–9	-0.3	30.6	0.1	25.8	6/29/2011	97.02	646.8	2	0.72
5–20	-0.7	30.2	0.2	25.4	6/29/2011	96.39	656.1	1	-3.95
5–10	-0.4	30.5	0.1	25.7	6/29/2011	96.35	658.8	2	-2.92
5–11	-0.4	30.5	0.1	25.8	6/29/2011	96.36	666.9	2	1.95
5–22	-0.5	30.4	0.2	25.6	6/29/2011	97.21	678.3	2	1.83
5–23	-0.9	30.0	0.2	25.2	6/29/2011	97.17	689.1	1	-0.79
5.5–1	-1.3	29.6	0.3	26.4	2/14/2014	97.70	699.5	1	-5.83
5–14	-0.9	30.0	0.1	25.2	6/29/2011	96.52	700.0	1	-5.41
5–24	-0.8	30.1	0.2	25.3	6/29/2011	96.95	703.0	2	-0.73
5.5–2	-0.9	30.0	0.3	26.8	2/14/2014	98.10	710.3	2	3.55
5–16	-0.8	30.0	0.1	25.3	6/29/2011	96.63	720.4	2	1.65
5.5–3	-0.8	30.0	0.3	26.8	2/14/2014	97.70	723.7	1	1.72
5.5–3	-0.8	30.0	0.3	26.8	2/14/2014	97.70	730.5	1	-1.57
5–26	-1.0	29.9	0.2	25.1	6/29/2011	96.45	731.8	1	-1.55
5–28	-1.1	29.8	0.2	25.0	6/29/2011	97.35	752.5	1	3.17
5.5–5	-0.5	30.4	0.3	27.2	2/14/2014	98.50	753.0	2	2.86
5.5–7	-0.6	30.3	0.3	27.1	2/14/2014	97.40	759.6	2	2.56
5.5–6	-0.2	30.7	0.3	27.5	2/14/2014	97.30	762.8	1	2.86
5.5–8	-0.7	30.2	0.3	27.0	2/14/2014	97.80	773.7	2	-4.64
5–29	-0.6	30.2	0.2	25.4	6/29/2011	96.17	776.7	1	-4.83
5.5–9	-0.5	30.4	0.3	27.2	2/14/2014	97.00	783.8	2	2.91
5.5–10	-0.4	30.5	0.3	27.3	2/14/2014	97.90	792.8	2	3.67
5.5–11	0.1	31.0	0.3	27.8	2/14/2014	95.30	840.6	1	0.46
6–2	0.0	30.9	0.3	27.6	2/14/2014	96.00	848.7	1	1.70
5.5–12	-0.3	30.7	0.3	27.4	2/14/2014	95.40	849.6	1	1.16
6–3	0.0	30.9	0.3	27.6	2/14/2014	95.00	858.3	1	0.44
5.5–13	-0.4	30.5	0.3	27.2	2/14/2014	96.20	866.4	1	3.01
6–4	-0.1	30.8	0.3	27.6	2/14/2014	95.40	875.6	1	1.75
5.5–14	0.0	30.9	0.3	27.6	2/14/2014	96.60	885.1	1	-3.61
6–6	-0.8	30.0	0.4	27.0	2/14/2014	98.20	922.0	2	0.99
6–7	-1.0	29.9	0.4	26.8	2/14/2014	98.40	937.9	1	-1.70
7–1	-0.5	30.4	0.3	27.6	2/14/2014	97.50	938.8	1	-1.34
6–8	-0.3	30.6	0.4	27.5	2/14/2014	98.30	950.9	1	3.56
7–3	0.1	31.0	0.3	28.2	2/14/2014	97.30	953.3	1	2.56
6–9	-0.6	30.3	0.4	27.2	2/14/2014	98.60	964.4	2	-3.09
7–4	-0.7	30.2	0.3	27.5	2/14/2014	97.20	971.9	2	-2.15
7–5	-0.4	30.5	0.3	27.7	2/14/2014	97.20	984.5	1	4.88
6–11	-0.4	30.5	0.4	27.4	2/14/2014	98.90	992.6	1	-2.44
7–6	-0.4	30.5	0.3	27.8	2/14/2014	97.60	994.1	1	-3.60
7–7	-0.5	30.4	0.3	27.6	2/14/2014	97.40	1005.5	1	-1.61
6–13	-0.3	30.6	0.4	27.5	2/14/2014	99.50	1020.3	1	3.47
7–9	0.1	31.0	0.3	28.2	2/14/2014	96.70	1021.3	1	3.16
6–14	-0.4	30.5	0.4	27.4	2/14/2014	99.50	1025.1	2	1.32
7–10	-0.5	30.4	0.3	27.6	2/14/2014	97.20	1034.7	1	-1.13
8–1	-0.4	30.5	0.4	27.8	2/14/2014	98.70	1038.0	2	-1.08
7–11	-0.6	30.3	0.2	27.6	2/14/2014	98.20	1041.0	1	-2.69
6–16	-0.5	30.4	0.4	27.3	2/14/2014	99.40	1042.5	2	-2.41
8–2	-0.5	30.4	0.4	27.8	2/14/2014	97.90	1047.6	1	0.42
7–12	0.1	31.0	0.2	28.2	2/14/2014	97.90	1048.8	1	1.35
8–3	-0.5	30.4	0.4	27.8	2/14/2014	98.90	1058.1	1	2.00
8–4	-0.9	30.0	0.4	27.3	2/14/2014	98.40	1067.4	1	-3.70
7–14	-0.3	30.6	0.2	27.9	2/14/2014	98.50	1072.2	1	-2.21
7–15	-0.4	30.5	0.2	27.7	2/14/2014	99.10	1077.1	1	-0.51
7–16	-0.3	30.6	0.2	27.8	2/14/2014	98.60	1085.5	1	1.27
8–6	-1.1	29.8	0.4	27.1	2/14/2014	99.00	1089.1	1	1.47
8–7	-1.4	29.5	0.4	26.8	2/14/2014	99.80	1099.9	1	-2.15
7–18	-0.8	30.1	0.2	27.3	2/14/2014	98.30	1104.7	1	-1.97
8–8	-0.3	30.6	0.4	27.9	2/14/2014	98.90	1112.5	1	-2.00
7–19	-0.7	30.2	0.2	27.5	2/14/2014	98.70	1117.3	1	-0.15
8–9	-0.8	30.1	0.4	27.5	2/14/2014	98.00	1125.1	1	1.94
7–20	-0.3	30.6	0.2	27.9	2/14/2014	98.40	1128.7	2	1.83
9–2	-0.4	30.5	0.1	26.9	2/15/2014	98.80	1153.1	1	-3.63
8–12	-0.2	30.7	0.4	27.3	2/15/2014	98.70	1157.0	1	-2.10
8–13	-0.2	30.7	0.4	27.3	2/15/2014	98.50	1166.3	1	4.64
9–3	0.0	30.9	0.1	27.3	2/15/2014	98.40	1169.6	2	2.89
9–4	0.2	31.1	0.1	27.5	2/15/2014	98.10	1175.6	1	3.03
8–15	-0.1	30.8	0.4	27.4	2/15/2014	98.30	1176.2	1	2.26
9–5	-0.1	30.8	0.1	27.3	2/15/2014	98.60	1179.2	1	2.67
8–16	-0.6	30.3	0.4	26.9	2/15/2014	98.90	1190.6	1	-3.25
9–6	-0.4	30.5	0.1	27.0	2/15/2014	98.30	1193.6	1	-4.05
9–7	-0.3	30.6	0.1	27.1	2/15/2014	97.80	1199.3	1	-0.53
8–17	-0.5	30.4	0.4	27.0	2/15/2014	99.00	1199.6	2	-0.86
9–8	-0.5	30.4	0.1	26.9	2/15/2014	99.00	1214.1	2	1.21
8–18	0.0	30.9	0.4	27.4	2/15/2014	100.00	1215.0	1	1.77
8–19	0.3	31.2	0.4	27.8	2/15/2014	99.90	1225.8	1	-6.13
9–9	0.6	31.5	0.1	28.0	2/15/2014	99.90	1226.7	1	-6.46
8–20	0.2	31.1	0.4	27.7	2/15/2014	98.70	1229.1	2	-4.94
10–1	0.4	31.3	0.3	27.7	2/15/2014	98.70	1234.2	1	-1.46
9–10	0.9	31.8	0.1	28.3	2/15/2014	99.60	1235.1	1	-1.02
10–2	0.4	31.3	0.3	27.7	2/15/2014	99.10	1241.1	1	3.50
9–11	0.1	31.0	0.3	27.5	2/15/2014	99.00	1242.0	1	2.87
10–3	0.2	31.1	0.3	27.5	2/15/2014	99.20	1247.4	2	2.01
9–13	-0.3	30.6	0.3	27.1	2/15/2014	98.80	1258.2	2	-0.05
10–5	-0.6	30.3	0.3	26.7	2/15/2014	98.30	1268.2	1	1.61
9–14	-0.7	30.2	0.3	26.6	2/15/2014	98.80	1269.7	1	1.23
10–6	-0.7	30.2	0.3	26.6	2/15/2014	98.40	1282.3	1	-0.19
9–16	-1.2	29.7	0.3	26.1	2/15/2014	97.30	1285.9	2	-0.82
10–7	-0.4	30.5	0.3	27.0	2/15/2014	98.00	1288.0	1	-0.04
9–18	-0.8	30.1	0.3	26.5	2/15/2014	99.10	1304.8	1	-1.80
10–9	0.0	30.9	0.3	27.3	2/15/2014	98.00	1309.9	1	-1.43
9–19	-0.7	30.2	0.3	26.6	2/15/2014	98.50	1311.7	1	-3.00
9–20	-0.5	30.4	0.3	26.9	2/15/2014	98.90	1321.3	2	1.02
10–11	0.4	31.3	0.3	27.7	2/15/2014	98.70	1331.6	2	-0.85
10–12	0.1	31.0	0.3	27.3	2/15/2014	98.50	1337.3	2	0.32
10–13	-0.1	30.8	0.3	27.2	2/15/2014	98.70	1342.7	1	3.25
10–14	-0.6	30.3	0.3	26.7	2/15/2014	99.10	1352.9	2	-3.02
10–15	0.1	31.0	0.3	27.4	2/15/2014	98.30	1365.2	2	1.26
10–17	0.4	31.3	0.3	27.7	2/15/2014	98.50	1376.3	1	1.45
11–1	0.2	31.1	0.4	27.5	2/15/2014	98.10	1381.1	1	2.03
10–18	-0.2	30.7	0.3	27.0	2/15/2014	98.60	1382.0	1	1.27
12–1	0.5	31.5	0.3	28.0	2/15/2014	98.00	1384.0	1	-0.19
11–2	-0.1	30.8	0.4	27.2	2/15/2014	98.80	1384.1	2	-0.30
10–19	-0.1	30.8	0.3	27.2	2/15/2014	98.90	1388.3	1	-5.04
10–21	0.3	31.2	0.4	27.6	2/15/2014	99.30	1396.9	1	-2.10
10–20	0.1	31.0	0.3	27.4	2/15/2014	98.10	1398.0	2	-2.02
12–2	0.0	30.9	0.3	27.4	2/15/2014	98.10	1398.6	1	-1.42
11–3	-0.3	30.6	0.4	27.1	2/15/2014	98.40	1399.8	1	-0.08
12–3	0.5	31.4	0.3	27.9	2/15/2014	97.90	1409.5	1	3.24
10–22	0.0	30.9	0.4	27.4	2/15/2014	98.60	1411.5	2	3.04
11–4	0.3	31.3	0.4	27.7	2/15/2014	97.90	1413.3	1	2.66
11–6	0.3	31.2	0.4	27.6	2/15/2014	98.00	1421.7	1	-1.09
12–4	0.3	31.2	0.3	27.7	2/15/2014	97.80	1427.0	1	0.64
10–24	-0.4	30.5	0.4	26.9	2/15/2014	100.40	1430.1	1	1.69
12–5	0.4	31.3	0.3	27.8	2/15/2014	97.50	1437.9	1	-1.22
11–7	-1.0	29.9	0.3	26.4	2/15/2014	99.30	1438.8	2	-1.57
11–8	0.2	31.1	0.3	27.6	2/15/2014	99.20	1444.5	1	-4.31
12–6	0.7	31.6	0.3	28.1	2/15/2014	97.90	1450.8	1	-0.77
11–9	0.5	31.4	0.3	27.9	2/15/2014	98.70	1455.9	2	3.31
11–10	0.6	31.6	0.3	28.1	2/15/2014	98.60	1458.4	2	3.19
12–7	-0.3	30.6	0.3	27.1	2/15/2014	97.50	1480.7	1	-0.73
11–13	-0.5	30.4	0.3	26.9	2/15/2014	99.30	1481.5	2	-0.41
12–8	-0.4	30.5	0.3	27.0	2/15/2014	98.40	1482.1	1	-0.24
11–14	0.0	30.9	0.3	27.4	2/15/2014	98.80	1497.1	1	1.41
11–15	0.2	31.1	0.3	27.6	2/15/2014	98.20	1504.0	2	1.13
11–16	-0.2	30.7	0.3	27.2	2/15/2014	98.8	1506.7	2	1.23
11–18	-0.3	30.6	0.3	27.1	2/15/2014	98.7	1523.3	2	0.77

Analysis names refer to Transect#-Pit#.

** Conversion from VSMOW and corrected to aragonite (-0.98) δ^18^O_VPDB_ = (δ^18^O_VSMOW_− 30.91)/1.03091.

* Value adjusted by bracketing standard and corrected to aragonite.

^λ^ Value reported vs. VSMOW δ^18^O_raw_ = {(^18^/_16_ measured/0.00200520)-1}*1000

^£^ Precision for δ^18^O_VPDB_ measurements derived from 2 times standard deviation of eight bracketing standards.

^€^ Yield relative to the mean yield of 8 bracketing standards.

^₭^ Distance values in micrometers perpendicular to visible banding to center of analysis pit.

^ Normalized CLFM grey scale value from averaged line of pixels parallel to growth banding in CLFM image for pit location with lowess regression removed to correct for trends in brightness across and between images. Low values are dark portions of growth bands and high values are bright portion.

#### δ^18^O variation and CLFM fluorescence

SIMS oxygen isotope analysis of the prismatic layer of both samples shows no correlation between measured oxygen isotope ratios and CLFM fluorescence at pit locations. For the wild-caught sample, both Pearson’s product-moment correlation (p = 0.17, cor = 0.15) for all regular pits (C1 and C2) and Spearman’s rank order correlation (p = 0.24, ρ = 0.13) are of low magnitude and not statistically significant. Likewise, for the aquarium-reared sample, both correlation tests are of low magnitude and non- significant (Pearson cor = 0.11, p = .58 and Spearman = 0.047, p = .844).

#### δ^18^O variation within bands

**Fig 5:**
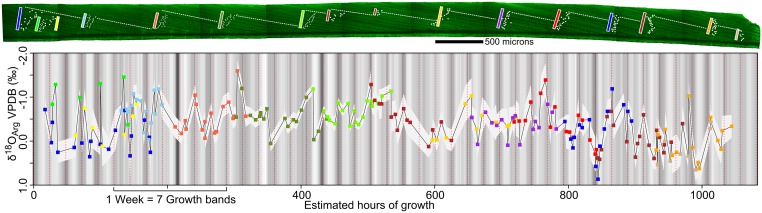
SIMS results and CLFM brightness for transects perpendicular to banding in the outer prismatic layer of the wild-caught *Nautilus macromphalus* (AMNH 105621). SIMS analysis and CLFM imaging was done on this 8 mm long portion of vacuum roasted and polished *Nautilus macromphalus* shell. The background grayscale is based on the residual CLFM brightness after lowess regression to correct for differences in brightness within and between images. Oxygen isotope ratios are plotted against hours of growth, assuming that each dark-light-dark cycle is 24 hours. The most recently precipitated shell is at time 0. Overlapping transects are color coded to show where correlation across the shell was carried out to produce the composite record. Transect locations are highlighted on the CLFM image of the outer prismatic layer. Correlation shows agreement within instrumental precision between transects correlated across the shell. There is considerable oxygen isotope variability within daily individual growth bands and across multiple bands. A higher resolution map of the shell surface is available in the supplementary information (S1 Fig).

**Fig 6:**
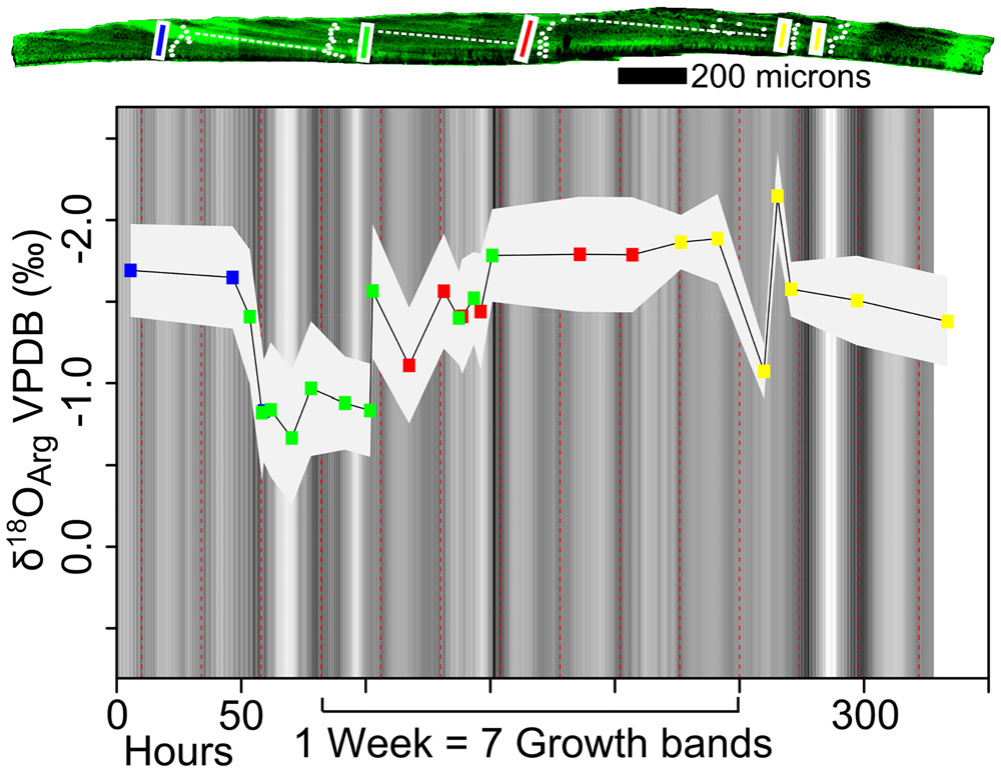
SIMS results and CLFM brightness for transects perpendicular to banding in the outer prismatic layer of the aquarium-reared *Nautilus belauensis* (AMNH 102555). SIMS analysis and CLFM imaging was done on this 3-mm long portion of vacuum roasted and polished *Nautilus belauensis* shell. The background grayscale is based on the residual CLFM brightness after lowess regression to correct for differences in brightness across and between images. Oxygen isotope ratios are plotted against hours of growth, assuming that each dark-light-dark cycle is 24 hours and the scales of the x and y axes match those of Fig 5. The most recently precipitated shell is at time 0. Overlapping transects are color coded to show where correlation across the shell was done to produce the composite record. Transect locations are highlighted on the CLFM image of the outer prismatic layer. Correlation shows general agreement within instrumental precision between transects correlated across the shell. Finding variability in this sample is particularly interesting because it is one of three individuals that is often cited as an example of *Nautilus* precipitating oxygen in isotopic equilibrium with seawater [12]. The positive shift in δ^18^O_Arg_ that takes place over several days is attributed to evaporation of ~8 liters of water from the 80 L incubation tank in which this individual was living [45,47,76]. A higher resolution map of the shell surface is available in the supplementary information (S2 Fig).

SIMS oxygen analyses vary within the bands visible in CLFM images (Figs 5 and 6). The amount of variation captured by analysis is dependent on the pit spacing within the band and the placement of the pits. Bands with more pits (3 or more) are more likely to have a large range in δ^18^O_Arg_. In the wild-caught sample, variation beyond instrument precision (2SD) is observed in 26 of the 39 bands that contain 2 or more analysis pits with an average within band range in δ^18^O_Arg_ of 0.8‰ and a maximum range of 1.5‰ (Fig 5). Within the aquarium-reared sample, only 2 of the 4 bands with three or more pits had ranges outside of expectations for instrumental precision with an average range of 0.6‰ and a maximum range of 1‰ (Fig 6). Correlation between transect locations shows nearly equal δ^18^O values between closely spaced pits on the traverse perpendicular to banding (Figs 5 and 6).

#### δ^18^O variation across bands

Cycles are present across several bands in both the wild-caught sample and the aquarium-reared sample (Figs 5 and 6). The wild-caught sample has several cycles that occur across 2–3 bands. The aquarium-reared sample has one long- term cycle across 8 bands where an increase in δ^18^O is slow over 5 bands, and then there is a rapid decrease over 3 bands (from 275–50 h, Fig 6). The range of δ^18^O_Arg_ measured in the wild-caught sample was from 0.6 to -1.9‰ (VPDB). In the aquarium-reared sample the range was 1‰ smaller than that of the wild-caught sample (measured values from -1 to -2.5‰ VPDB).

## Discussion

### Growth banding

Growth bands that are observed in CLFM, UV, and plain light images (Figs 1 and 3) in both *Nautilus* are interpreted to occur daily. Each band is delimited by a repeated pattern in the amount of organic matter relative to aragonite in the shell, which is most clearly imaged in CLFM as repeated patterns in the luminescence of the shell (Figs 1, 3, 4, 5 and 6). Similar growth banding on a daily timescale has been observed in gastropods reared in aquaria [28]. The apertural growth rates calculated from growth band width range from 104 to 308 μm/day in the wild-caught *Nautilus macromphalus* and from 65 to 160 μm/day in the aquarium-reared *Nautilus belauensis*. The width of growth banding within the prismatic layer visible by CLFM in *Nautilus* has not been previously reported in the literature; however, apertural growth rate has been [3,20,21,42,72–74,77,78]. Growth rates for *Nautilus* grown in aquaria range from 0 to 600 μm/day [42] with an average of 158 μm/day across nine studies, although abnormal shell growth in aquaria is common [21]. The similarity between growth rates determined from band width and published values suggest that the intraprismatic banding is daily in *Nautilus*. The trend across 7–8 growth bands toward higher δ^18^O values in the aquarium-reared sample (Fig 6) also suggests daily growth bands, because the duration of the pattern may from evaporative enrichment in the aquarium over one week as reported in the aquarium management procedure [45,47]. Shell growth across a wide range of depths can be inferred from the 2.5‰ δ^18^O range across the wild-caught sample, which is larger than ranges reported for sampling across entire shells [1,4,8,11]. Variation within growth bands of up to 1.5‰ indicate a range of temperatures of at least ~5°C, which can likely only be captured if growth is continuous in a mobile organism at the SIMS sampling resolution (~7 hours for 10 μm growth). If growth was not continuous across all depths or growth rate varied widely with temperature, δ^18^O should not vary within or across the sampled growth bands by as much as we have detected. However, because our analyses do include some amount of time-averaging, truncation or slowing of growth during extremely shallow or deep excursions cannot be excluded as a possibility. Because growth banding can be interpreted to be daily and growth rate variation within individual days cannot be reliably inferred, both Figs 5 and 6 have been scaled so that each dark—light—dark band is equal to 24 hours and growth rate is uniform within individual days.

### δ^18^O_Arg_ variability in *Nautilus macromphalus* and *Nautilus belauensis*

SIMS analysis of both the wild-caught sample and the aquarium-reared sample found δ^18^O_Arg_ variation within the outer prismatic layer of aragonite, but the character of variability is strikingly different between the two samples (Figs 5 and 6). The aquarium-reared *Nautilus belauensis* (AMNH 102555) has a prominent gradual increase in the δ^18^O_Arg_ values across ~7 growth bands and then an abrupt decrease over ~1 growth band (at 75 hours, Fig 6). This gradual increase can be explained by evaporative increase of δ^18^O [76] followed by a sudden decrease during recharge and replacement with lower δ^18^O water. This refill and replacement cycle occurred once weekly over approximately 8 hours to prevent shocking the *Nautilus*, with water coming from a salt-water well [45,47]. Experimental evaporation studies where the δ^18^O of residual liquid was measured after evaporation under controlled-humidity air (at 0, 20, 51% humidity, 19.6 to 20°C) suggest that ~10% evaporation from the tank will produce a shift of about +1‰ in the δ^18^O of aquarium water [76] on the timescale of 7 days. Subsequent refilling will cause a drop of comparable magnitude in ~8 hours because water is coming from a groundwater source that likely has a uniform δ^18^O. This evaporation and refilling is the main influence on the δ^18^O_Arg_ of the aquarium-reared sample, because temperature was kept constant at 22°C ±1°C (~0.4‰) [12,45,47]. Variation in the δ^18^O_Arg_ of the wild-caught *Nautilus macromphalus* (AMNH 105621) is greater within individual bands and of greater magnitude across fewer bands than the aquarium-reared individual (Figs 5 vs 6), which suggests a different mechanism for the formation of the variability.

### Oxygen isotope patterns and depth migration

It is widely accepted that changing habitat depth through weeks to years of an individual cephalopod’s lifetime can be interpreted from patterns in δ^18^O_Arg_ caused by the thermal gradient in the water column [1,2,5–9,11,12,79–82]. Variability in our new *in situ* measurements of δ^18^O_Arg_ can be interpreted as sub-daily depth-migration behavior if the following three criteria are met: 1) δ^18^O_Arg_ variation is largely decoupled from physiological variation (e.g. concentration of organic matter controlling CLFM fluorescence) 2) both high and low δ^18^O_Arg_ values are observed within individual bands (suggesting different depths within one day), and 3) the range of δ^18^O_Arg_ variation falls near or within expected values from depth constraints and water column conditions near New Caledonia (~20°C or ~4.0‰ between 0 to 750 m, Fig 2 [1,15]). These three criteria provide a consistent test for the presence or absence of the depth migration behavior in modern and fossil cephalopods. Testing all three criteria requires collecting a dataset that combines high precision *in situ* isotope analyses with a physical record of growth, but does not require shell to be precipitated in equilibrium, only a uniform vital effect that does not vary within days.

If δ^18^O_Arg_ covaries strongly with physiological variation, such as growth banding, then variation in δ^18^O_Arg_ could be explained as a vital effect that fractionates carbonate during precipitation, where varying rates of precipitation would be related to the ratio of organic matter to carbonate in the shell and therefore CLFM brightness [83]. However, in the wild-caught and aquarium reared *Nautilus*, δ^18^O_arg_ varies within individual growth bands but does not covary with CLFM brightness (Figs 5 and 6), which suggests independent causes for growth banding and δ^18^O_Arg_ variability. Daily growth banding is caused by a physiological rhythm that is paced by a circadian clock [29,30,43]. A physiological pattern is expected to be regular because it is not controlled by the presence or absence of food or predators, but is controlled by the physical mechanisms of shell formation, in this case the production of the organic matter that is incorporated into the shell during precipitation. A behavioral pattern is not expected to perfectly covary with the circadian clock because it is the product of response of the individual *Nautilus* based on stochastic external stimuli, such as the appearance of food or predators [84]. This response to external stimuli is apparent in existing telemetry data because individuals are not at the same depth at the same time on different days [17–19]. Aquaria studies of other cephalopods (*Octopus*) demonstrate idiosyncratic timing of the activity of individuals even in a nocturnal population [85]. In the wild-caught *Nautilus macromphalus* (AMNH 105621), growth banding visible in CLFM is a recorder of a physiological rhythm and measured δ^18^O_Arg_ is a recorder of migration through a water column with a large (~20°C) thermal gradient across possible living depths (0–750 m) (Fig 2). In the aquarium-reared *Nautilus belauensis* (AMNH 102555), growth banding records a physiological rhythm, and the δ^18^O_arg_ records changing aquarium conditions.

Oxygen isotope variation larger than instrumental precision (95% CI) within or across individual bands is observed in 27 of the 41 bands with two or more analyses in them (Fig 6). Variation beyond precision strongly suggests that this *Nautilus macromphalus* was not staying within constant temperature or δ^18^O water during shell precipitation. Continuous precipitation within the limits of sampling resolution (10-μm spot ~7.5 hours) is suggested by intermediate oxygen isotope values between low and adjacent high values (Fig 6). Cycles that are apparent across several bands may be due to time averaging from the SIMS pits, aliasing of the δ^18^O record due to pit spacing, and pit-time averaging, or they could be a real record of a pattern of depth migration. It is also possible that the complete range of δ^18^O_arg_ was not sampled due to slowed growth at either cold or hot temperatures [86].

To create a δ^18^O_arg_ range of 2.5‰ (0.9 to -1.6‰ VPDB) in the wild-caught sample, the *Nautilus macromphalus* must have crossed a temperature gradient of ~12°C based on Eq 1, assuming constant δ^18^O_SW_. When temperatures are calculated using a δ^18^O_SW_ value of 0.5‰, they range from 27.6 to 15.5°C. The warm temperature is high for reported seawater temperature values (high of 26.3 Fig 2, [15]), but it is still possible for the *Nautilus* to have experienced this temperature without immediately dying [42,87,88] and *Nautilus macromphalus* have been observed in shallow water (<10 m) near New Caledonia [87]. This temperature range suggests the individual crossed at least ~400 m depth assuming a change of 0.3°C/m. This range of depth change over the 45 days is similar to that observed in shorter observation intervals by remote telemetry [17–19] and photocapture traps [20]. The range in δ^18^O is larger than that observed in adult shells by conventional analysis techniques [1–6]

### Comparison to remote telemetry migration data

**Fig 7:**
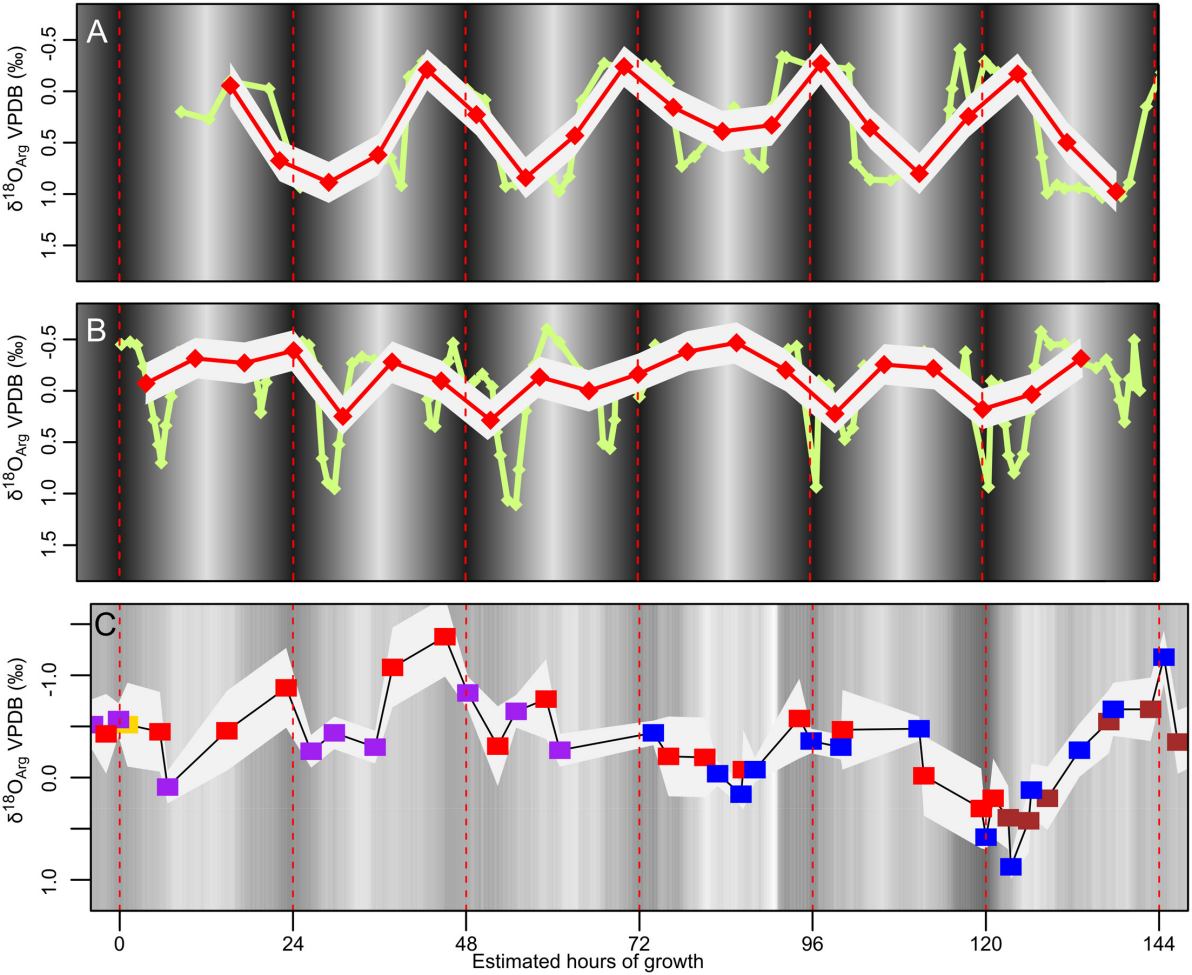
A comparison of SIMS measured δ^18^O_Arg_ to telemetry results that have been converted to δ^18^O_Arg_. Telemetry is done using ultrasonic transmitters that are attached to the dorsal shell of *Nautilus* and are monitored from boats or stationary underwater receivers [17–19]. SIMS results can be compared to telemetry records if water temperatures and δ^18^O are known, growth is continuous, and time averaging is calculated. Green lines in A and B are telemetry data converted to δ^18^O_Arg_ assuming a thermal gradient of 0.3°C/m, with a constant δ^18^O_sw_ of 0.5‰ (VSMOW) and using Eq 1. Overlying red lines are hypothetical SIMS data approximate time averaging from a 10-μm spot diameter SIMS spot assuming 35 μm/day growth. The gray background is the 2SD instrumental precision envelope around the SIMS analyses. A) Regular shallow-deep migrations observed in *Nautilus belauensis* off Palau [17] would likely leave a SIMS result with regular variability. B) Rapid excursions to depths at dawn and dusk, like those observed in *N*. *pompilius* off Osprey Reef, Australia [19] would leave less variability in the SIMS results due to the effects of time averaging. C) Actual SIMS results for the wild-caught *Nautilus macromphalus* from New Caledonia suggest that depth migration can be detected; however, there is not a regular migratory pattern like that observed at Palau. A different style of depth migration pattern, influenced by the local bathymetry or cover availability, could explain the differences between the telemetry data and the SIMS measurements. The δ^18^O_arg_ variation measured by SIMS in this study shows that the wild-caught sample spent significant amounts of time in both shallow and deep water, a behavior more similar to that observed by Ward *et al*. [17] and Carlson *et al*. [18] near Palau (fringing reef) than that observed by Dunstan *et al*. [19] near Osprey Reef (submerged atoll), Australia. Dunstan *et al*. [19] suggest that the behavioral difference between Osprey Reef and Palau is due to available hiding locations, and therefore it is possible that hiding locations on the fore-reef of the fringing reef near New Caledonia are less abundant than those on Osprey Reef. Differences in behavior could also be due to other factors of location or differences between species.

The pattern of oxygen isotope variability within bands can be compared to patterns of depth migration recorded by remote telemetry. Fig 7A illustrates the δ^18^O pattern calculated for one of the four individuals tracked by Ward *et al*. [17]. Dunstan *et al*. [19] reported more chaotic depth migratory patterns (Fig 7B). Time averaging from SIMS sampling would truncate a depth migration record and reduce the apparent amplitude of migration and duration at depth (Fig 7A and 7B) in the same way that time averaging of stable isotope sampling can truncate seasonal temperature variability in bivalves [86]. For instance, if an individual was stationary at a depth where δ^18^O_Arg_ was 1.0‰(VPDB) and then there was a short (<3 hour) migration to a depth where δ^18^O_Arg_ was 2.0‰ (VPDB), the difference between a point in the stationary depth and the migration depth would be ~0.4‰ assuming 7 hours/pit time averaging. Thus, a more chaotic migration pattern with very short excursions to extreme depths when measured by SIMS would have a greater dampening of δ^18^O_arg_ excursions because the domains showing highest or lowest δ^18^O_arg_ cannot be fully resulted by the 10 μm SIMS analysis pit (Fig 7B).

### Implications of sub-daily isotope sampling in Cephalopods

The capability to precisely measure sub-daily variation of δ^18^O within rapidly accreted biominerals presents new avenues for biological, paleobiological, and paleoecological research. Isotope analyses of well-preserved fossil cephalopods (i.e. nacreous microstructure preserved [89]) have been used to interpret ontogenetic habitat change and mean living depth [7–9,11,44,79,80,89–91]. This study suggests that estimating the activity and mobility of extinct cephalopods is possible by analyzing δ^18^O within growth bands in samples that preserve nacreous microstructure and the original mineralogy. These techniques can be used to test morphological hypotheses about the swimming ecology of ammonites [81,92,93]. Inferring depth migration behavior from δ^18^O in fossil material is a new approach for studies of marine ecology for situations where: 1) growth banding can be imaged to provide a physiological chronometer, and 2) a sufficient temperature or salinity gradient within ambient waters is present, which when analyzing fossil material can be determined by data from foraminifera or other benthic and planktonic organisms [91].

## Conclusions

SIMS analyses within visible growth banding of mollusk shells attain high spatial and analytical precision allowing us to measure a greater range in δ^18^O than that measured in adult shells using conventional analyses that average days to weeks of growth. Our new data record a depth-migratory signal in the δ^18^O values of the wild-caught *Nautilus macromphalus* shell. The width of growth banding visible by confocal laser fluorescence microscopy suggests that the bands are daily and the 10-μm diameter of SIMS pits used in this study yield an average time resolution of ~ 8 hours. Analysis of the aquarium-reared *Nautilus belauensis* shell suggests equilibrium precipitation, but also indicates that more controlled aquarium rearing studies are necessary. Combined, the results of this study serve as an example for the investigation of depth migration in fossil cephalopods. These results from *Nautilus* strongly suggest that high-resolution sampling across growth banding with SIMS can elucidate a signal of depth migration behavior by externally shelled cephalopods.

## Supporting Information

S1 Fig SEM and CLFM images of transects on *Nautilus macromphalus*, AMNH 105621. (PDF)

S2 Fig SEM and CLFM images of transects on *Nautilus belauensis*, AMNH 102555. (PDF)

S1 Table Table of all SIMS analyses including rejected values for sample *Nautilus macromphalus*, AMNH 105621. Complete data table of analyses in the outer prismatic layer of the wild-caught *Nautilus macromphalus* including bracketing standards and aragonite standard.
(XLSX)

S2 Table Table of all SIMS analyses including rejected values for sample *Nautilus belauensis*, AMNH 102555. Complete data table of analyses in the outer prismatic layer of the aquarium-reared *Nautilus belauensis* including bracketing standards and aragonite standard.
(XLSX)
