# Supplementary material for: Oxygen Isotope Variability within Nautilus Shell Growth Bands
Source: PLoS One. 2016 Apr 21;11(4):e0153890. doi: 10.1371/journal.pone.0153890 (PMC4839723; doi:10.1371/journal.pone.0153890)
Supplement: S1 Fig — (PDF) [file pone.0153890.s001.pdf]

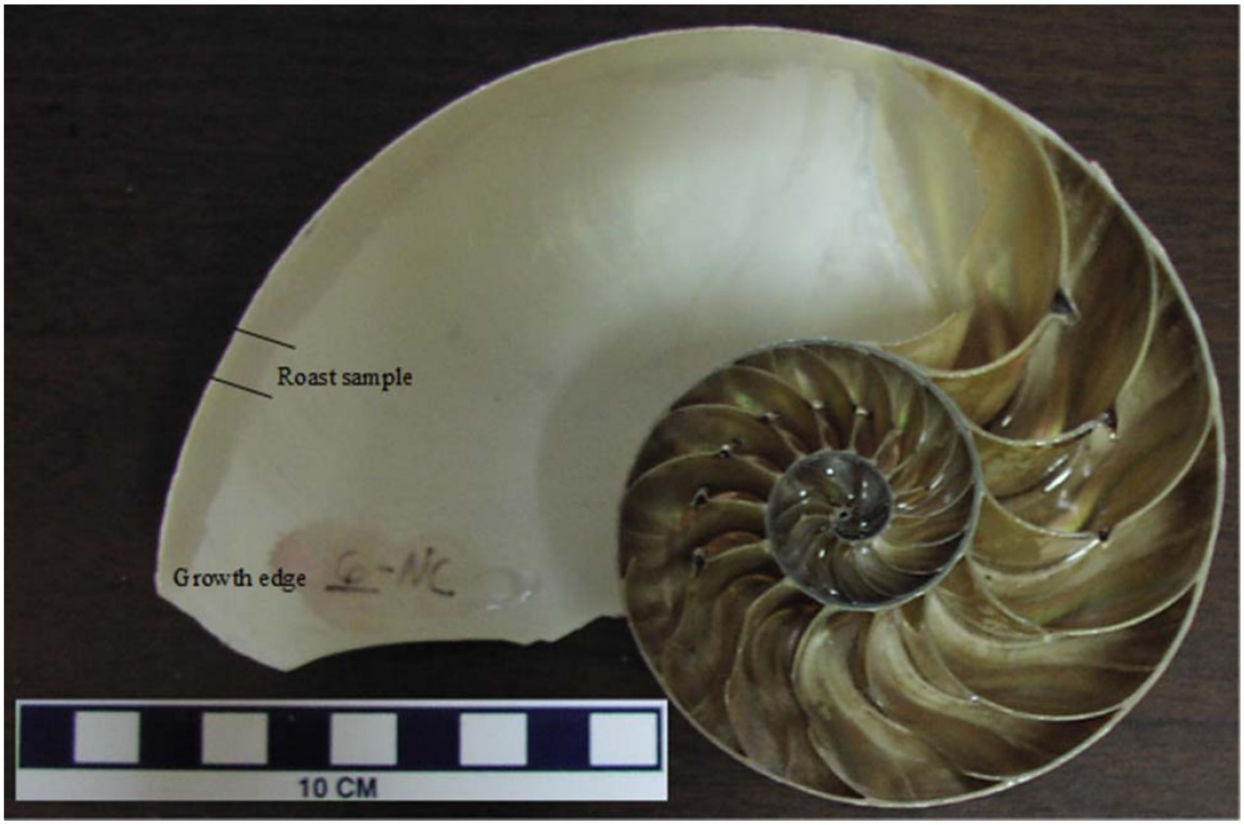

Image indicating where the roasted sample of the wild-caught Nautilus macromphalus was removed.

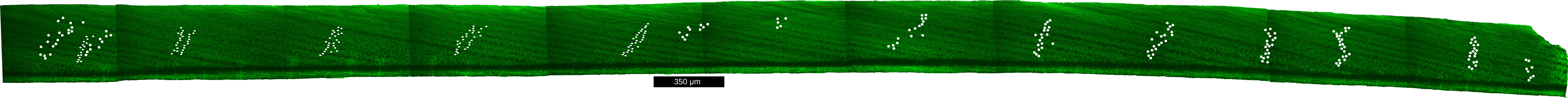
350 µm

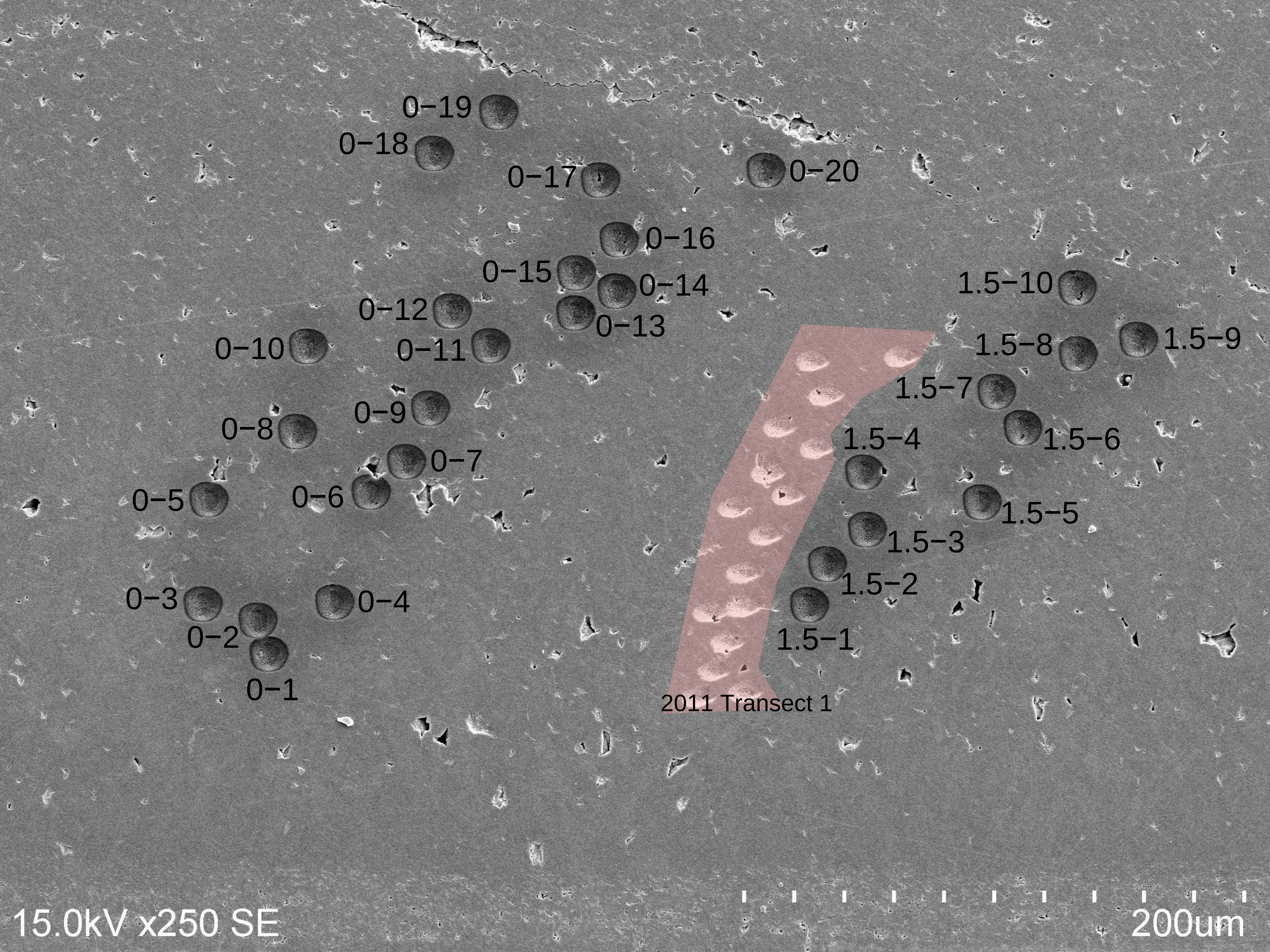
0−19
0−18
0−17
0−20
0−16
0−15
0−14
0−12
0−13
0−10
0−11
1.5−10
1.5−8
1.5−9
1.5−7
0−9
0−8
1.5−4
1.5−6
0−7
0−5
0−6
1.5−5
1.5−3
1.5−2
0−3
0−4
0−2
1.5−1
0−1
2011 Transect 1
15.0kV x250 SE
200um

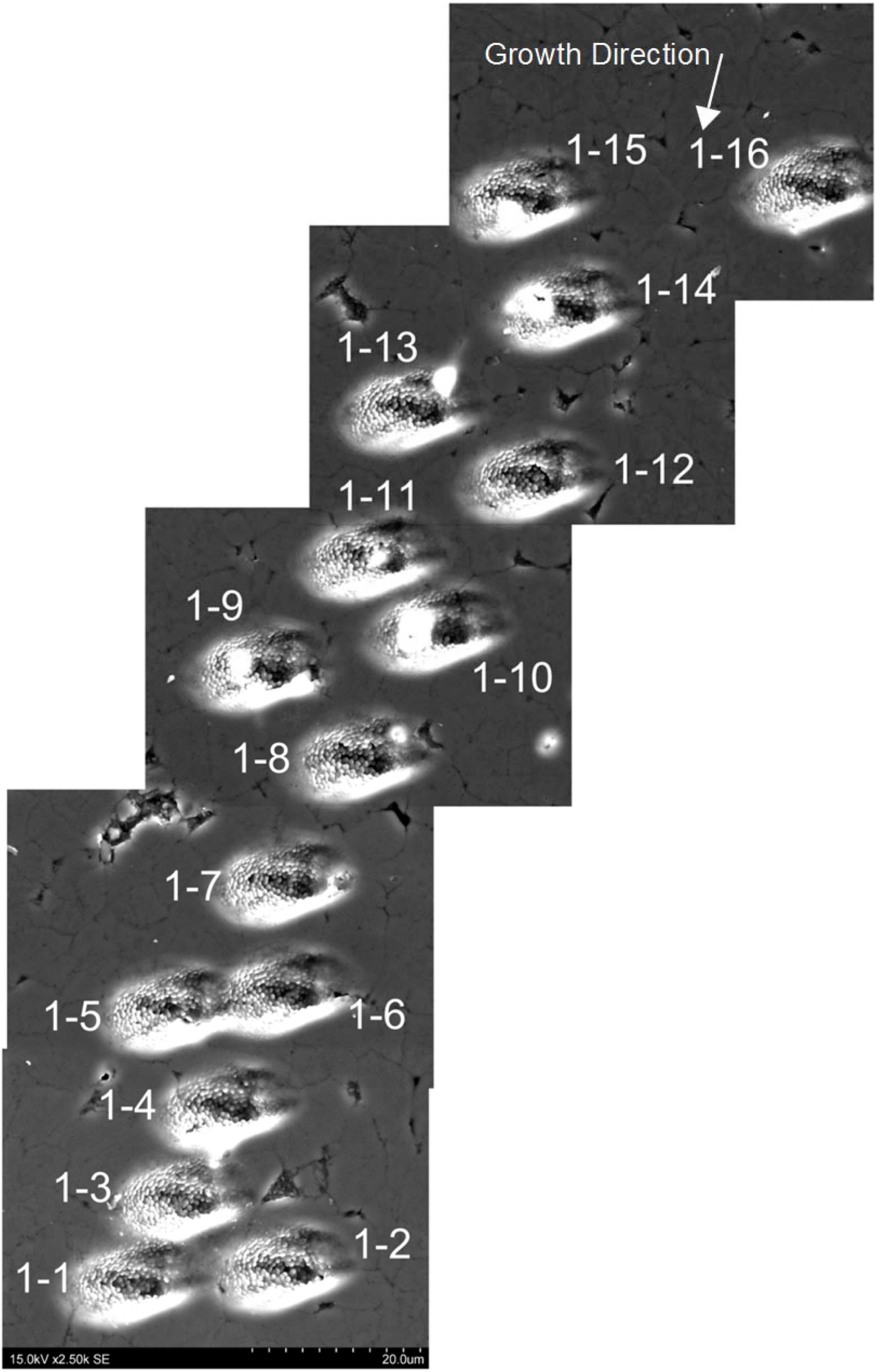
Growth Direction
1-15
1-16
1-14
1-13
1-12
1-11
1-9
1-10
1-8
1-7
1-5
1-6
1-4
1-3
1-2
1-1
15.0kV x2.50k SE
20.0um

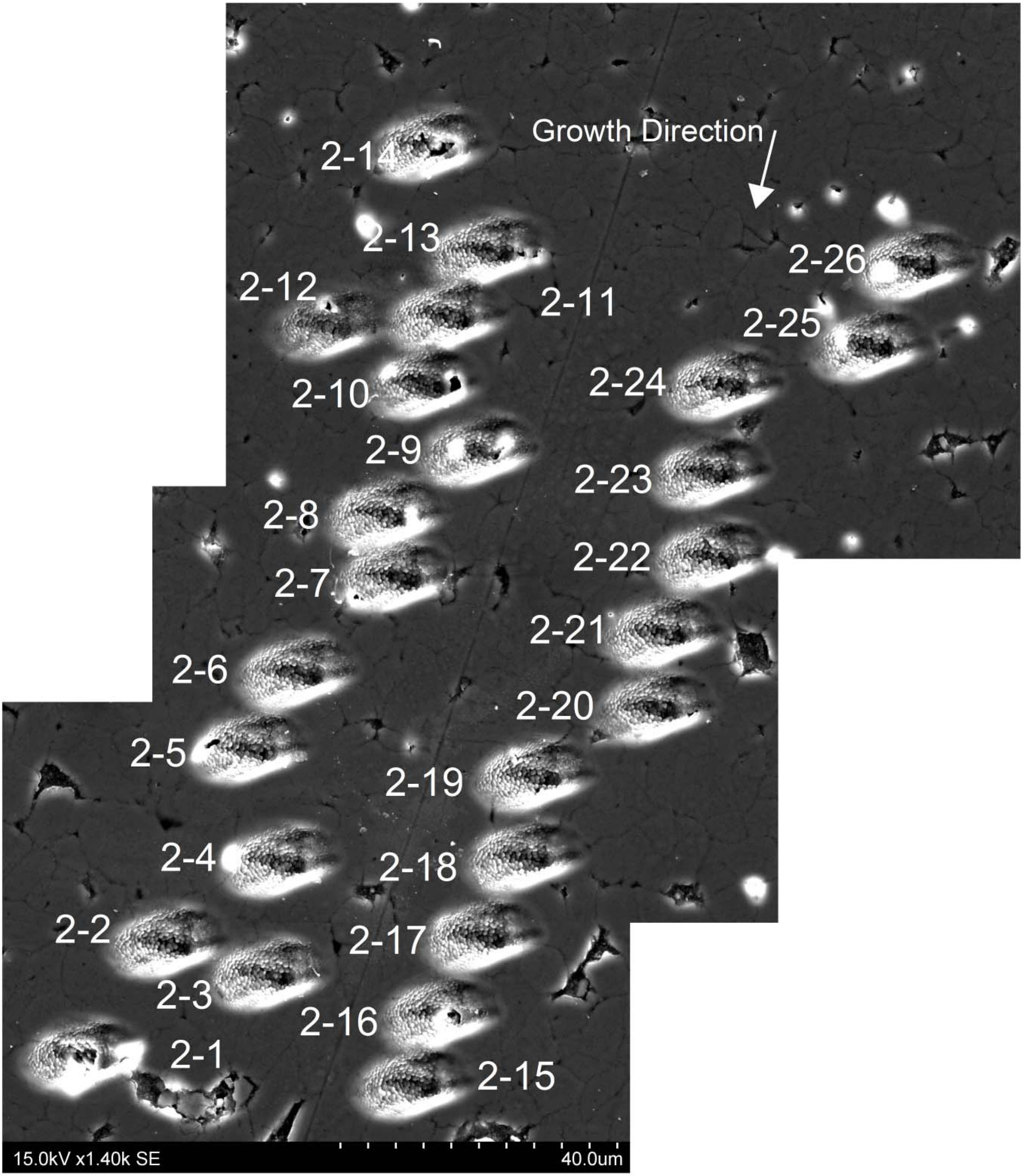
Growth Direction
2-14
2-13
2-12
2-11
2-26
2-25
2-24
2-10
2-9
2-23
2-8
2-22
2-7
2-21
2-6
2-20
2-5
2-19
2-4
2-18
2-2
2-17
2-3
2-16
2-1
2-15
15.0kV x1.40k SE
40.0um

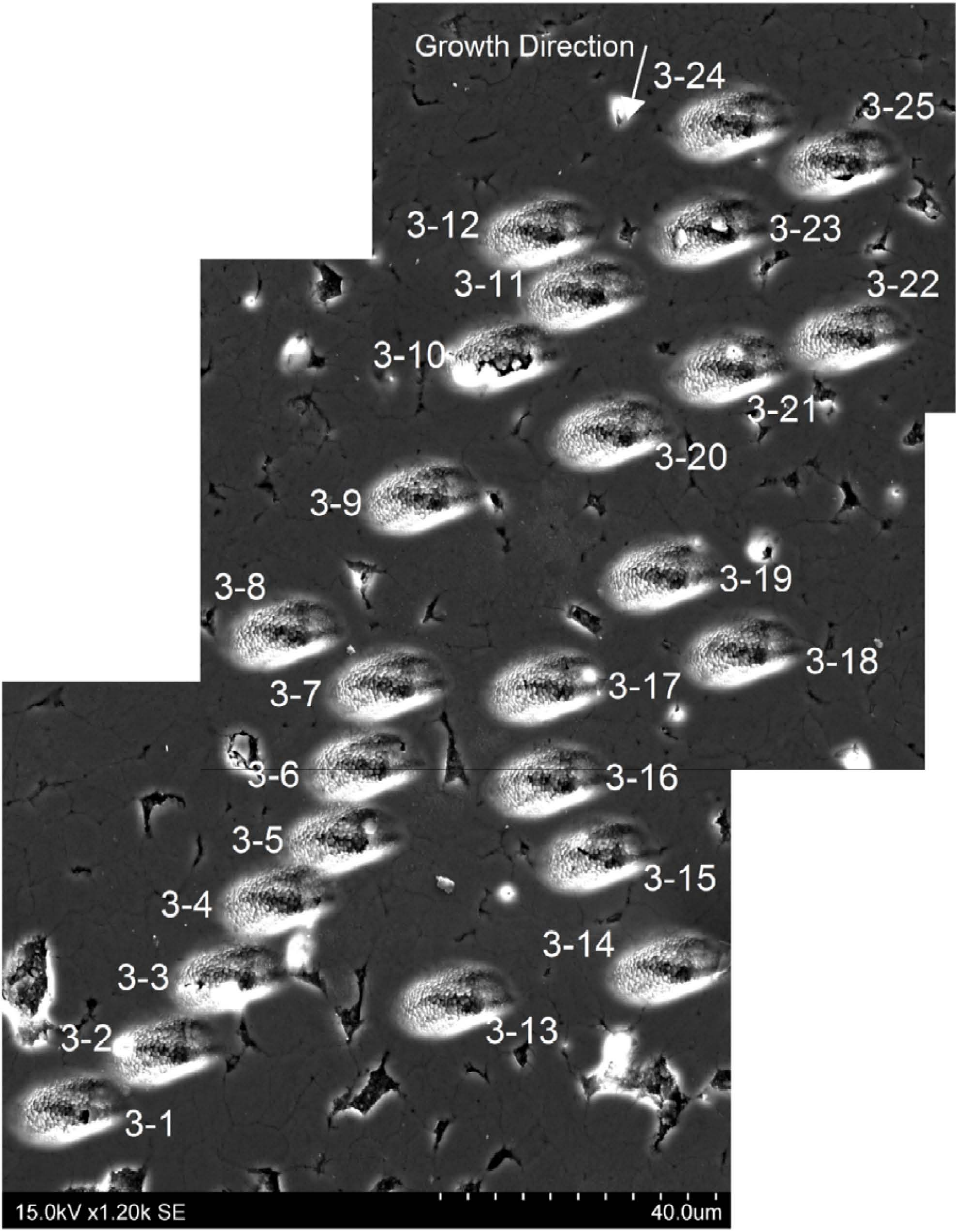

Growth Direction
3-24
3-25
3-12
3-23
3-11
3-22
3-10
3-21
3-20
3-9
3-19
3-8
3-18
3-7
3-17
3-6
3-16
3-5
3-15
3-4
3-14
3-3
3-13
3-2
3-1
15.0kV x1.20k SE
40.0um

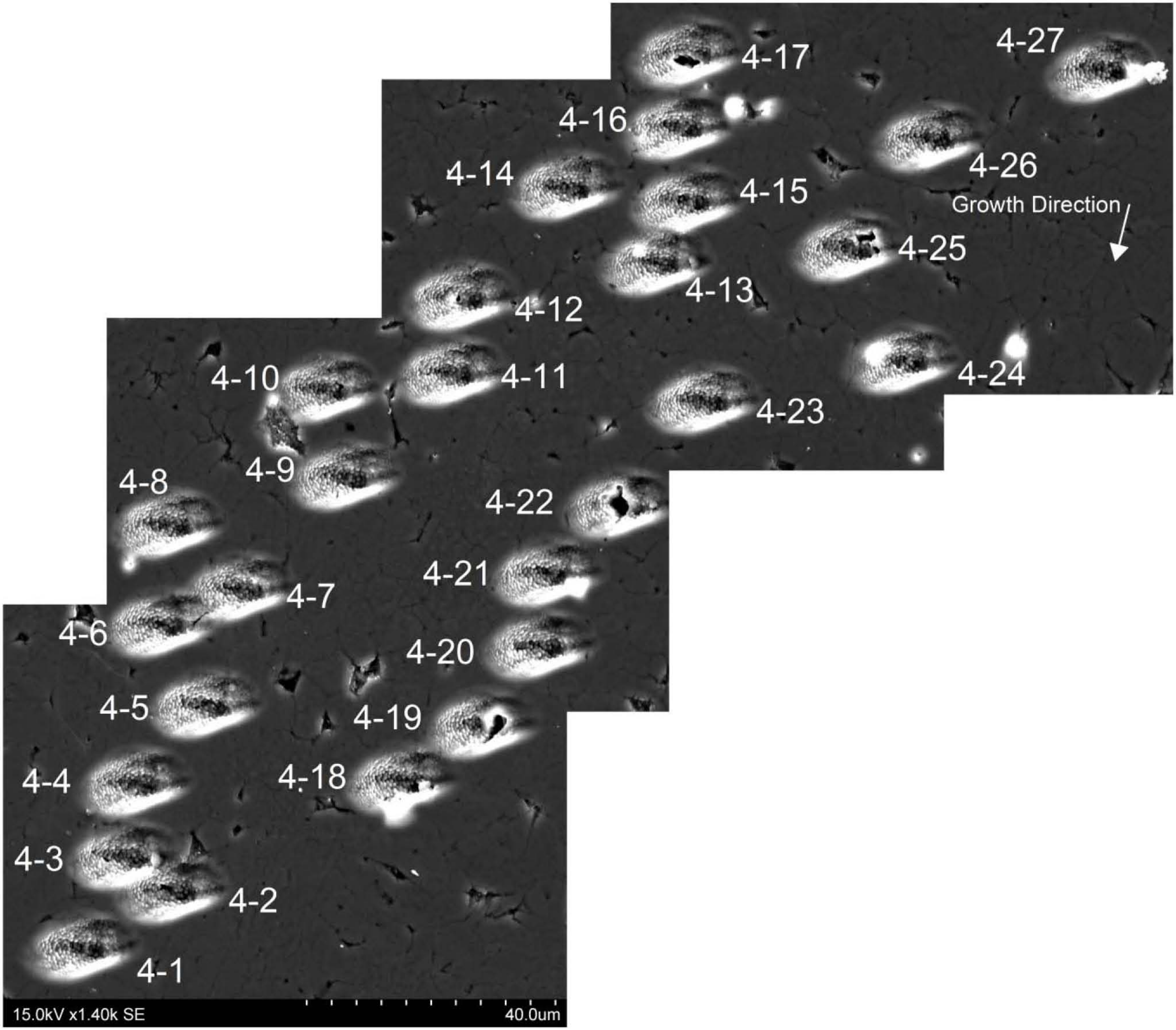
4-17
4-27
4-16
4-14
4-26
4-15
Growth Direction
4-25
4-13
4-12
4-10
4-11
4-24
4-23
4-8
4-9
4-22
4-21
4-7
4-6
4-20
4-5
4-19
4-4
4-18
4-3
4-2
4-1
15.0kV x1.40k SE
40.0um

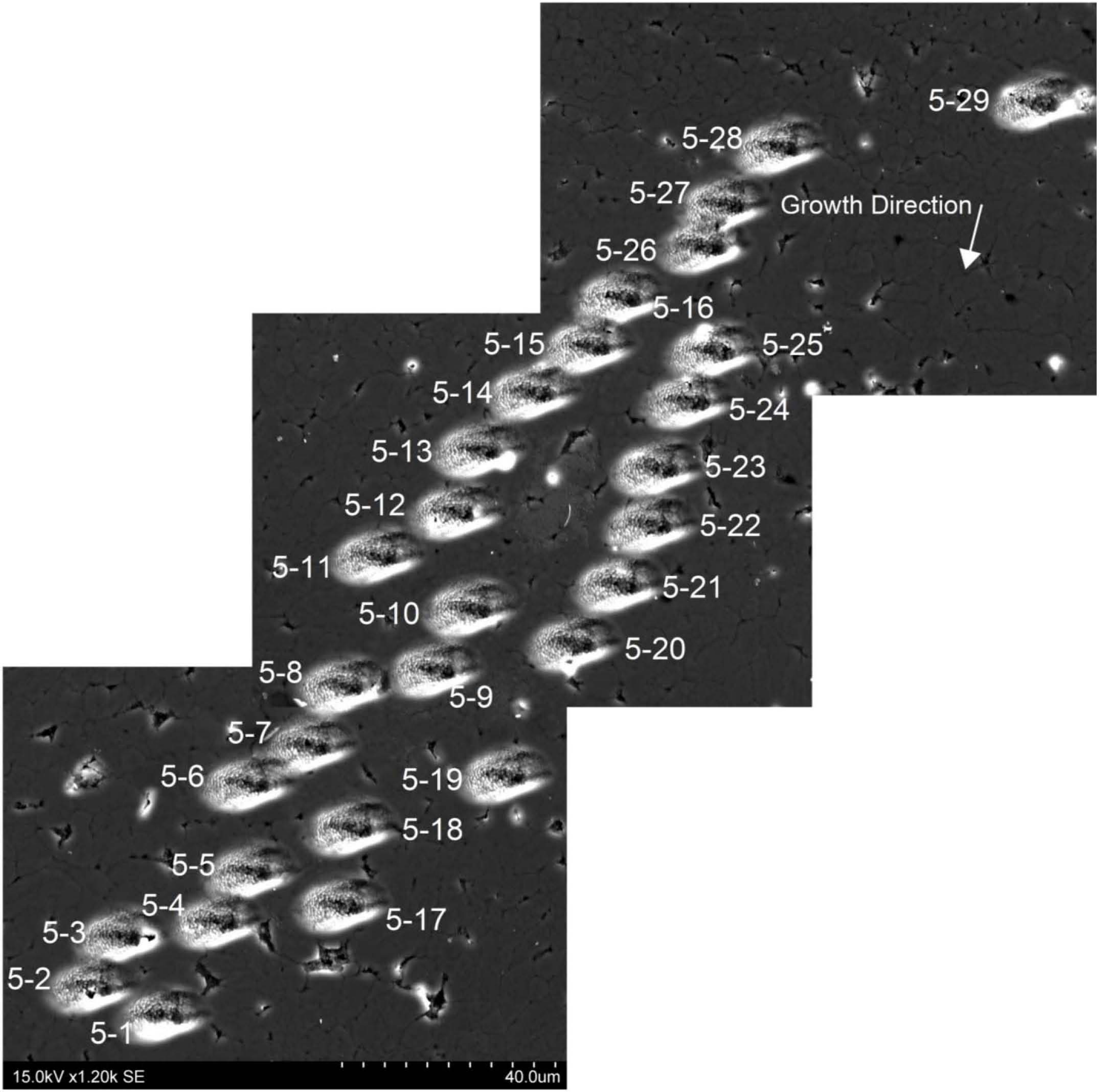
5-29
5-28
5-27
Growth Direction
5-26
5-16
5-15
5-25
5-14
5-24
5-13
5-23
5-12
5-22
5-11
5-21
5-10
5-20
5-8
5-9
5-7
5-6
5-19
5-18
5-5
5-4
5-17
5-3
5-2
5-1
15.0kV x1.20k SE
40.0um

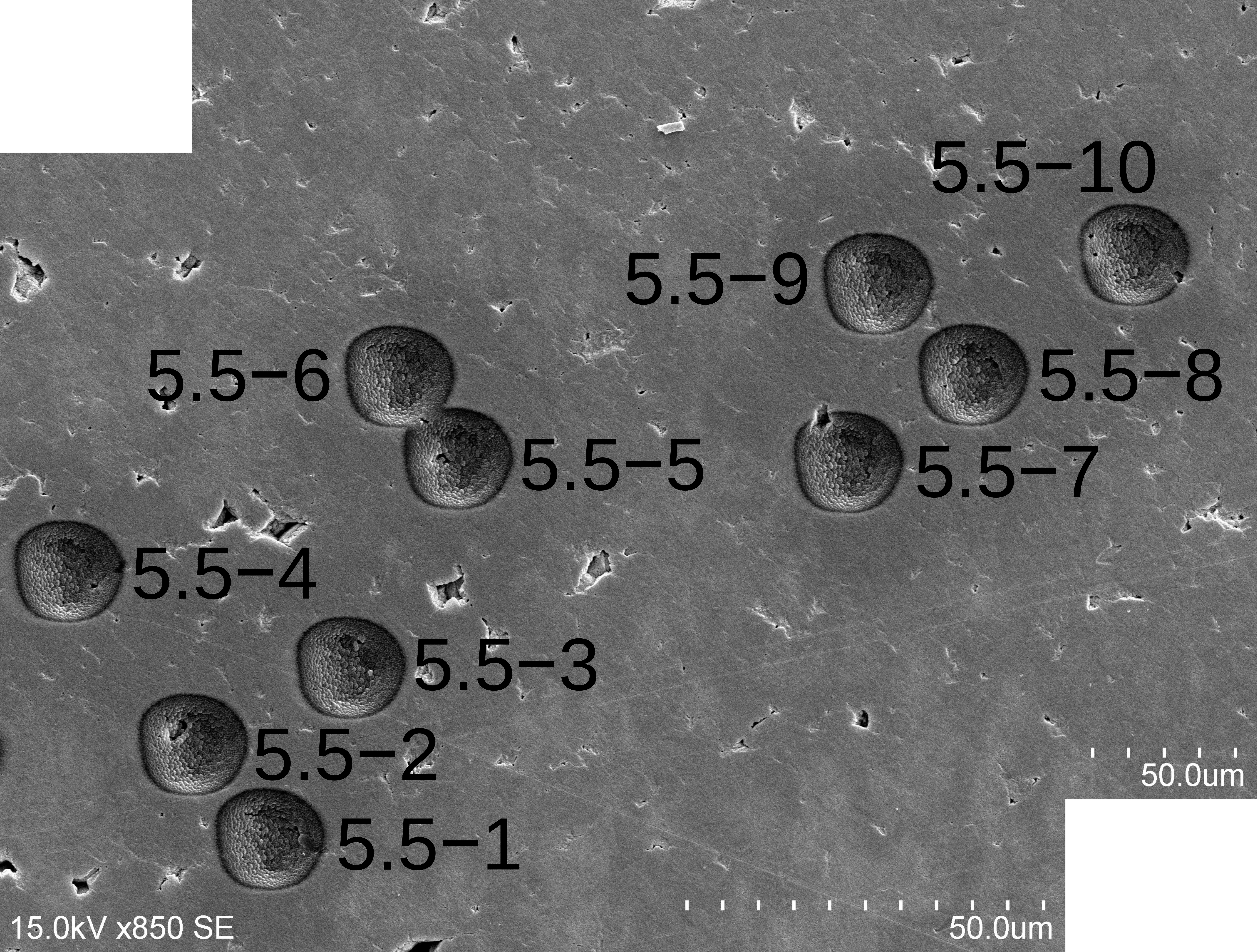

5.5−10
5.5−9
5.5−6
5.5−8
5.5−5
5.5−7
5.5−4
5.5−3
5.5−2
5.5−1
50.0um
15.0kV x850 SE
50.0um

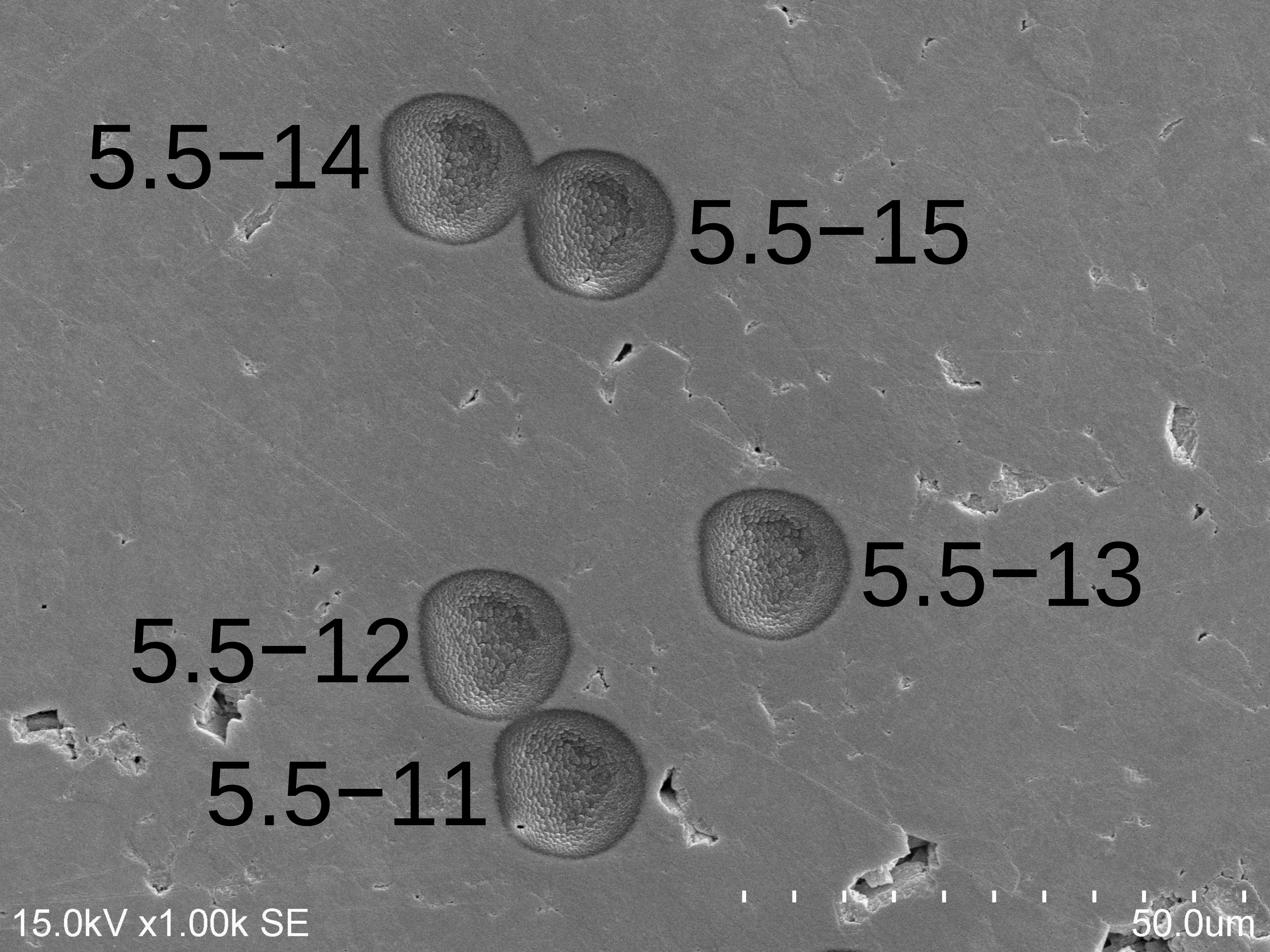
5.5–14
5.5–15
5.5–13
5.5–12
5.5–11
15.0kV x1.00k SE
50.0um

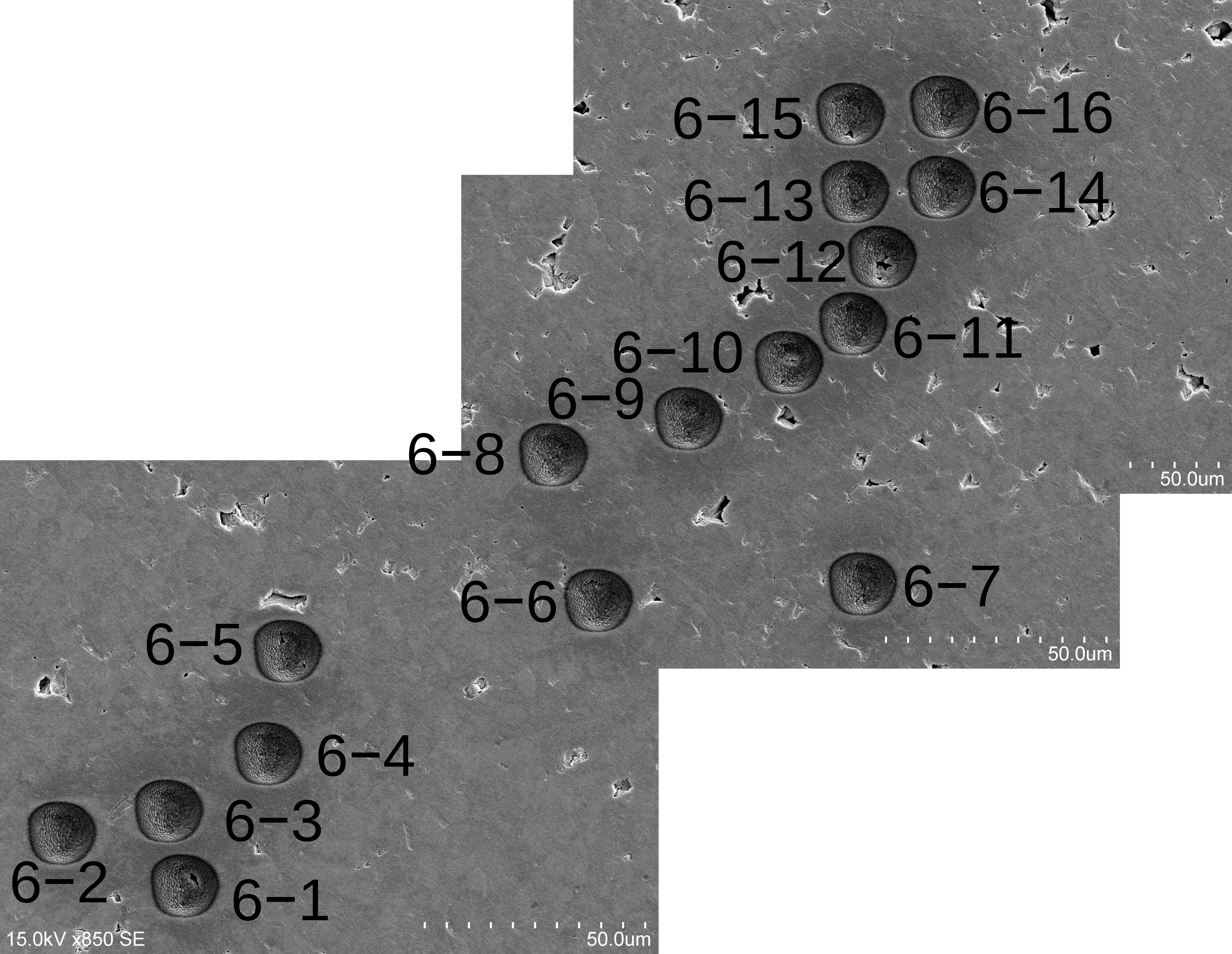
6−1
6−2
6−3
6−4
6−5
6−6
6−7
6−8
6−9
6−10
6−11
6−12
6−13
6−14
6−15
6−16
50.0um
50.0um
50.0um
15.0kV x850 SE

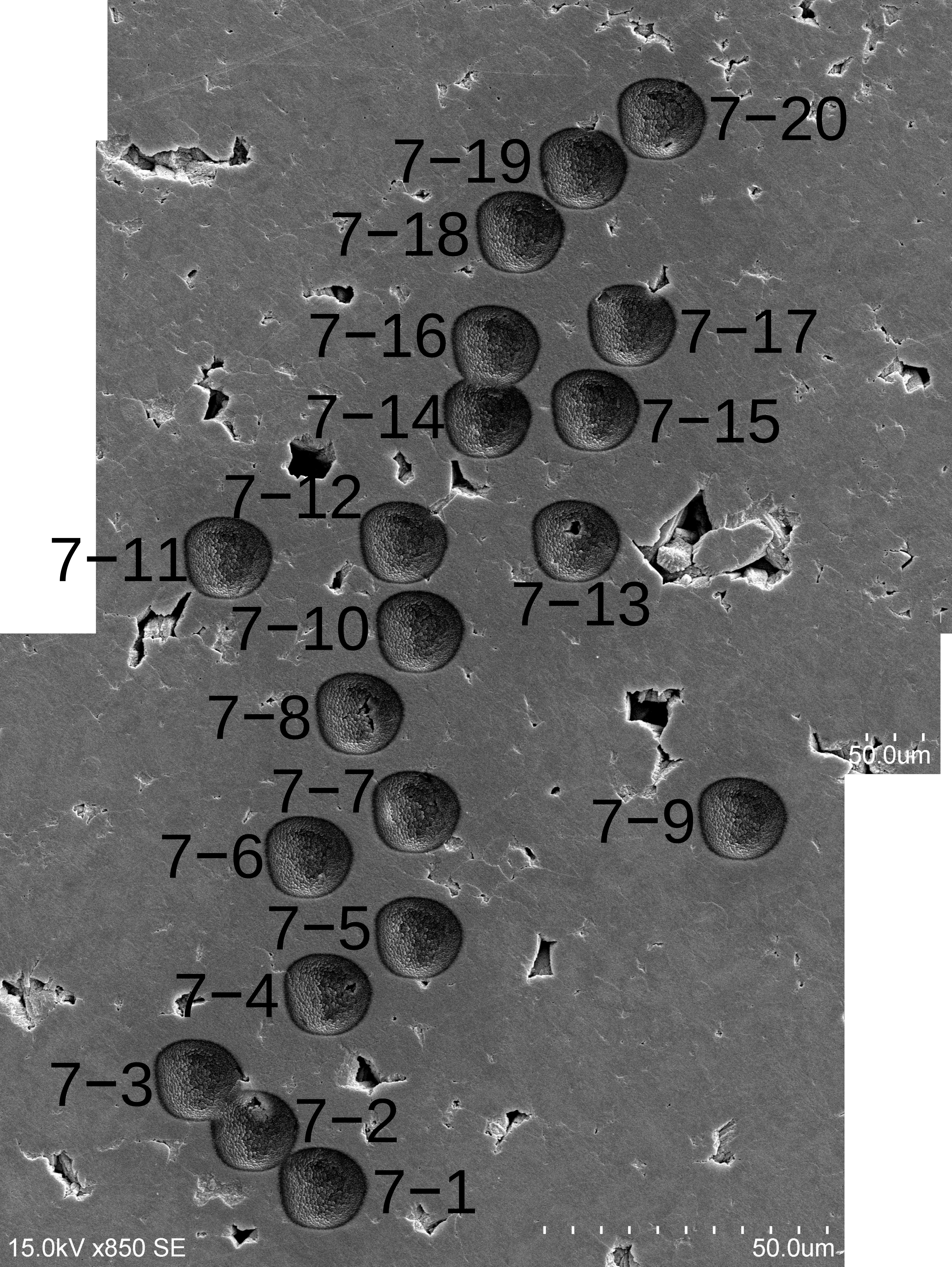
7−20
7−19
7−18
7−16
7−17
7−14
7−15
7−12
7−11
7−13
7−10
7−8
50.0um
7−7
7−9
7−6
7−5
7−4
7−3
7−2
7−1
15.0kV x850 SE
50.0um

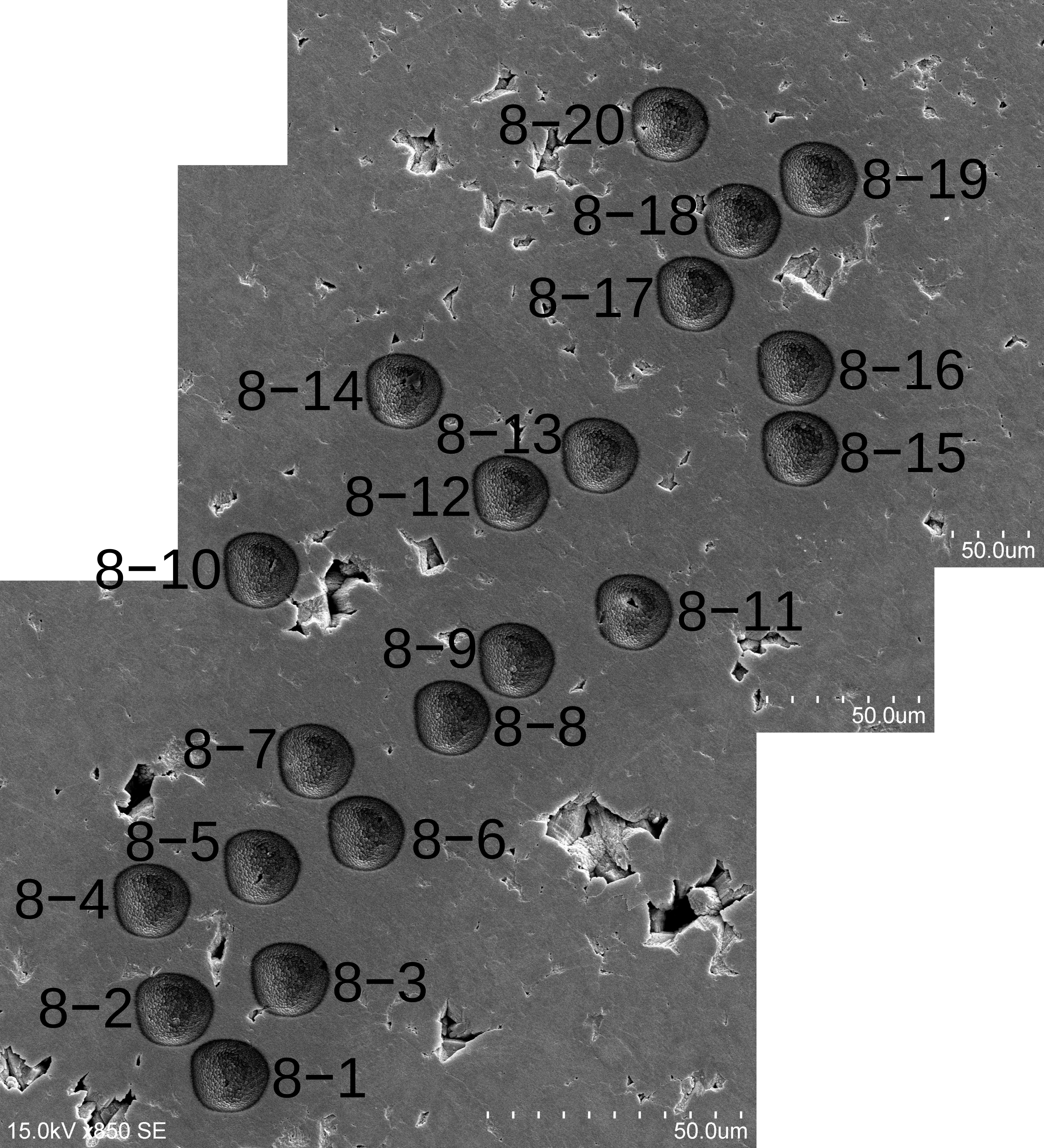
8−20
8−19
8−18
8−17
8−16
8−15
8−14
8−13
8−12
8−11
8−10
8−9
8−8
8−7
8−6
8−5
8−4
8−3
8−2
8−1
50.0um
50.0um
15.0kV x850 SE
50.0um

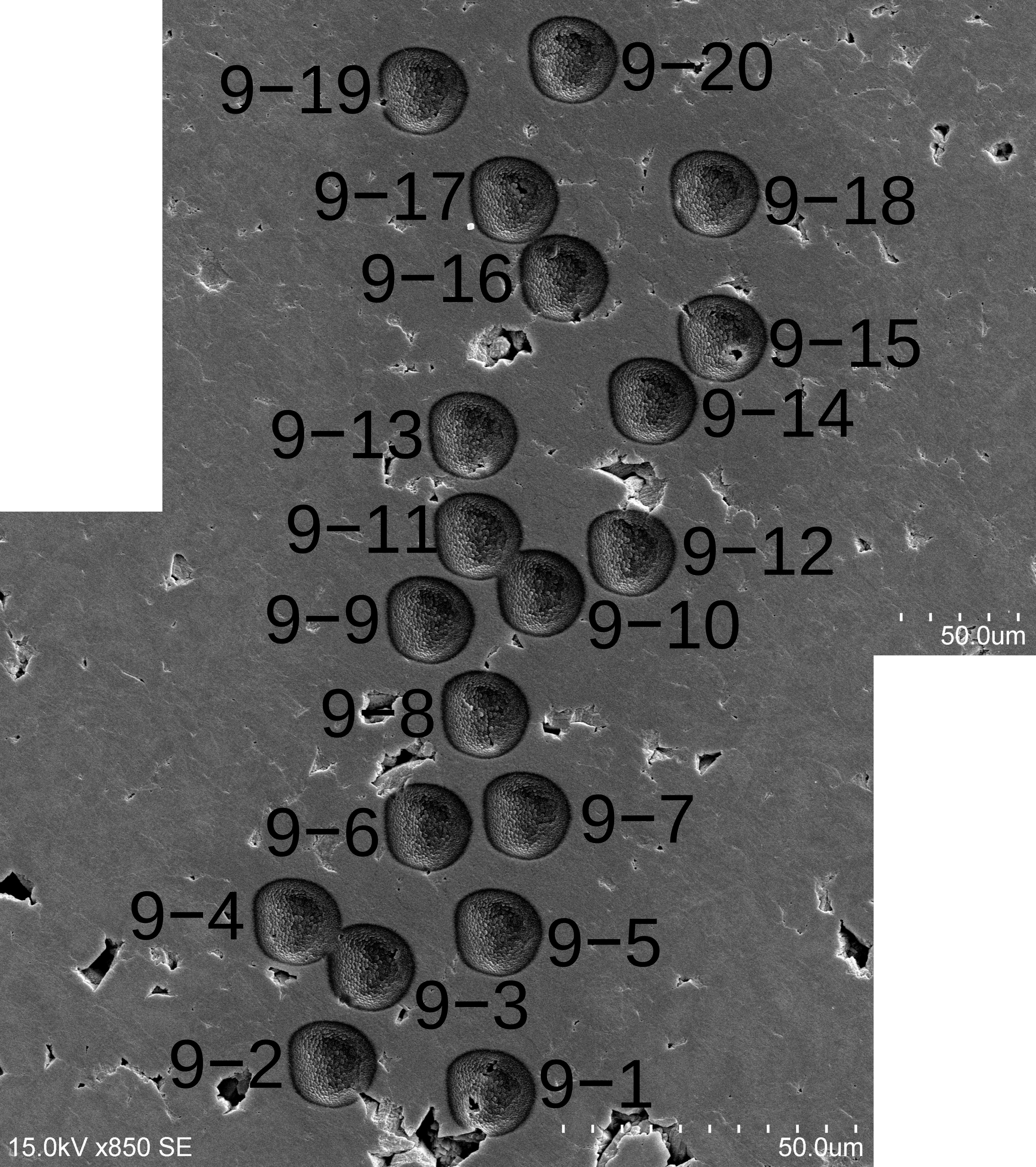
9−20
9−19
9−17
9−18
9−16
9−15
9−14
9−13
9−11
9−12
9−9
9−10
50.0um
9−8
9−7
9−6
9−4
9−5
9−3
9−2
9−1
15.0kV x850 SE
50.0um

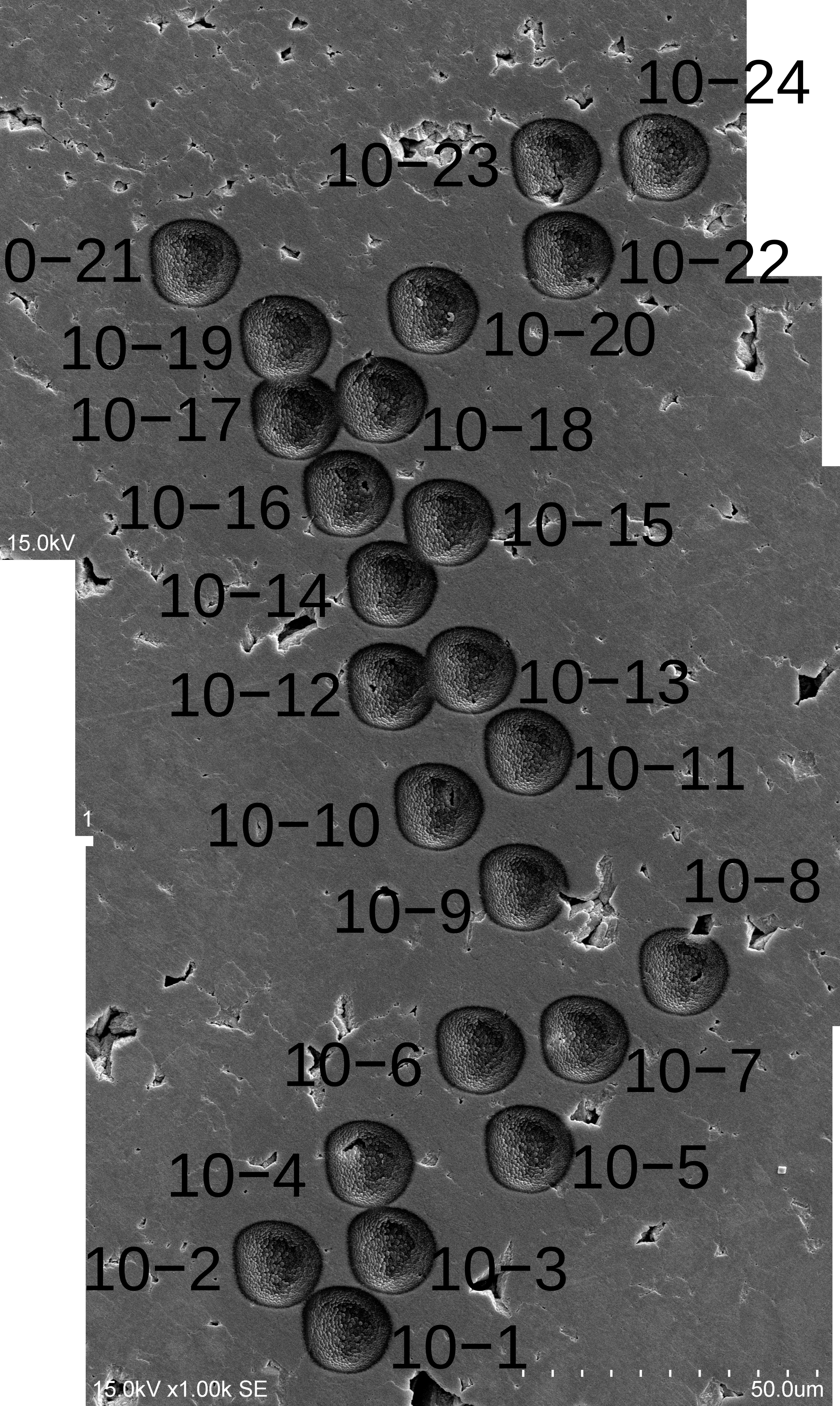
10−24
10−23
0−21
10−22
10−19
10−20
10−17
10−18
10−16
10−15
15.0kV
10−14
10−12
10−13
10−11
1
10−10
10−9
10−8
10−6
10−7
10−4
10−5
10−2
10−3
10−1
15.0kV x1.00k SE
50.0um

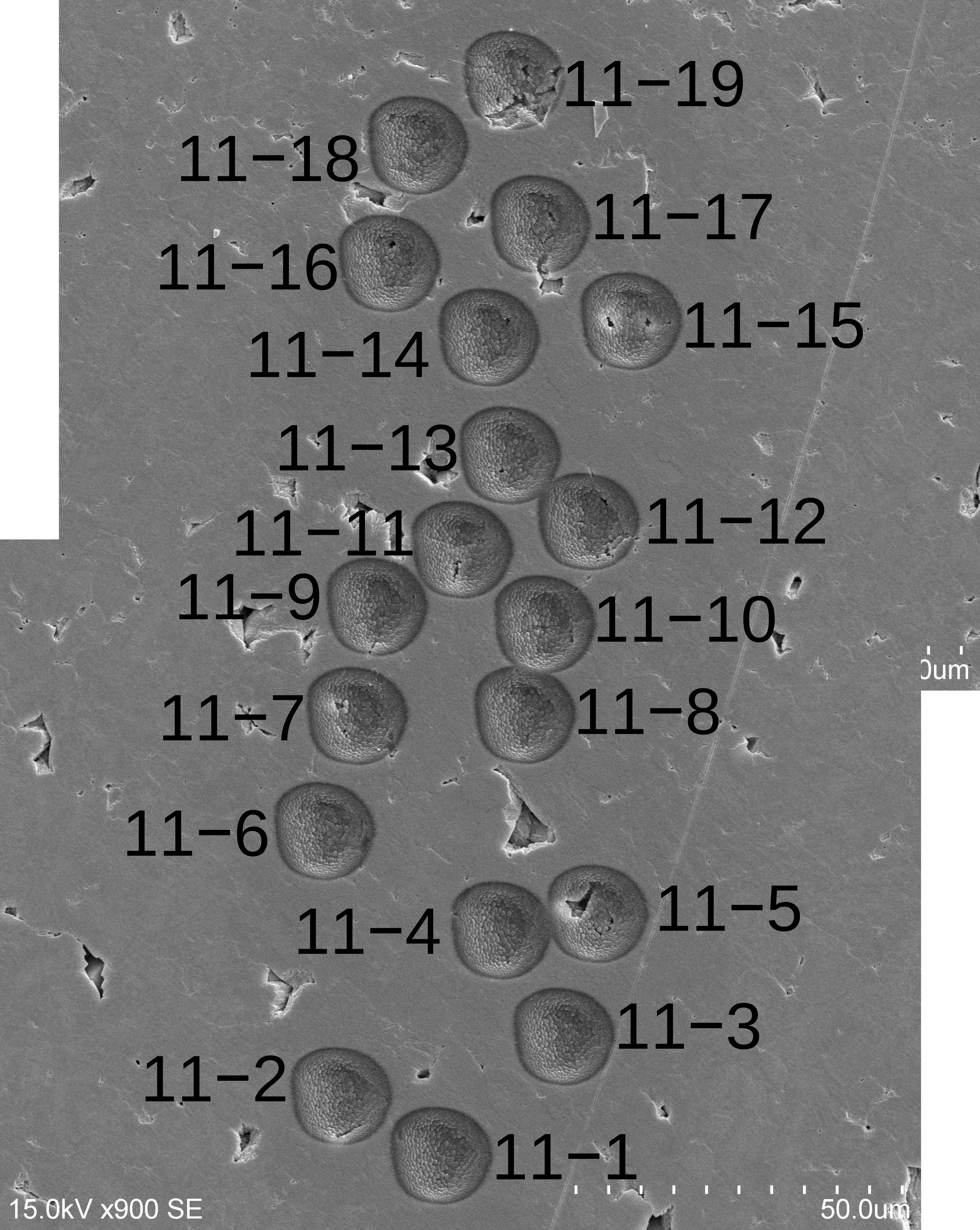
11−19
11−18
11−17
11−16
11−15
11−14
11−13
11−12
11−11
11−9
11−10
11−7
11−8
11−6
11−5
11−4
11−3
11−2
11−1
15.0kV x900 SE
50.0um

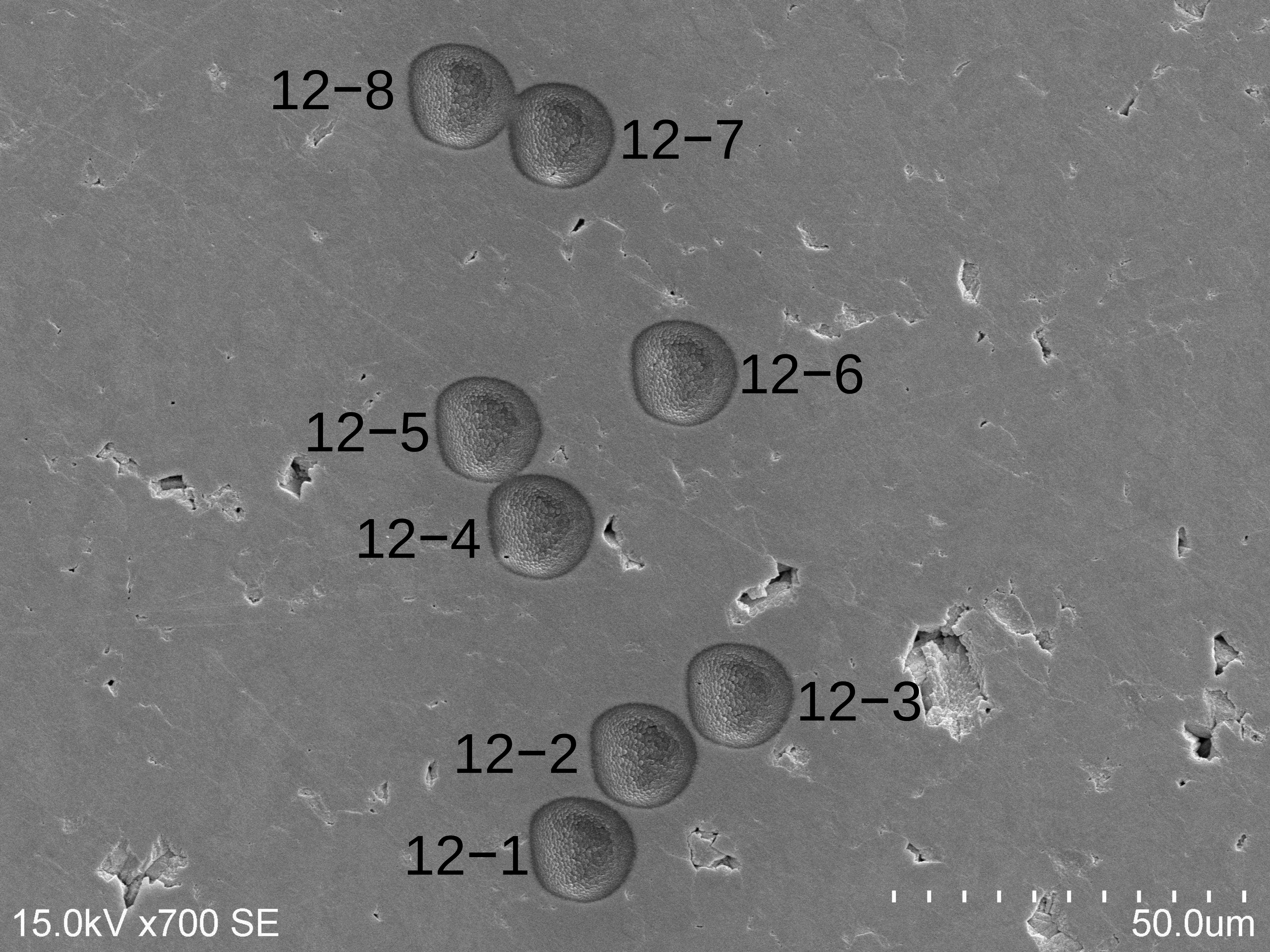
12−8
12−7
12−6
12−5
12−4
12−3
12−2
12−1
15.0kV x700 SE
50.0um
