# Supplementary material for: Oxygen Isotope Variability within Nautilus Shell Growth Bands
Source: PLoS One. 2016 Apr 21;11(4):e0153890. doi: 10.1371/journal.pone.0153890 (PMC4839723; doi:10.1371/journal.pone.0153890)
Supplement: S2 Fig — (PDF) [file pone.0153890.s002.pdf]

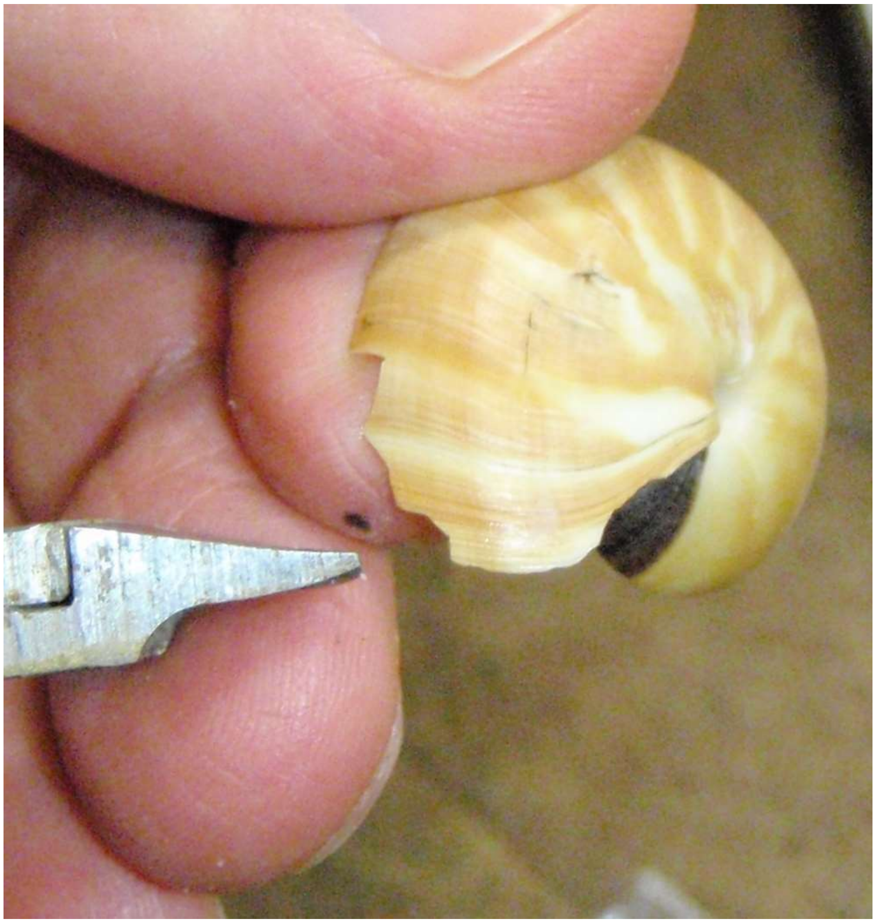

Indicating the location where the chip of *Nautilus belauensis* (AMNH 102555) was removed near the aperture.

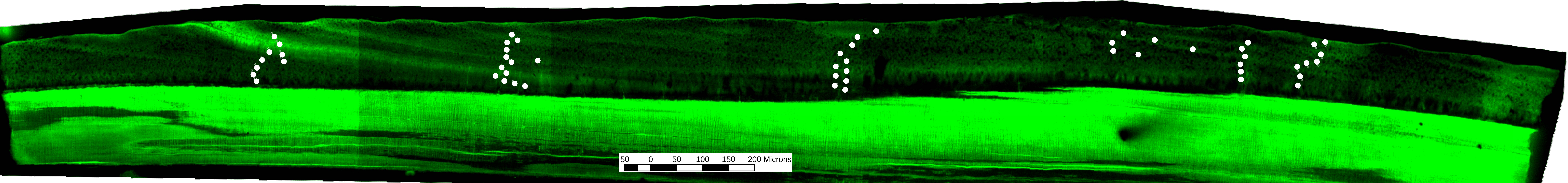
50 0 50 100 150 200 Microns

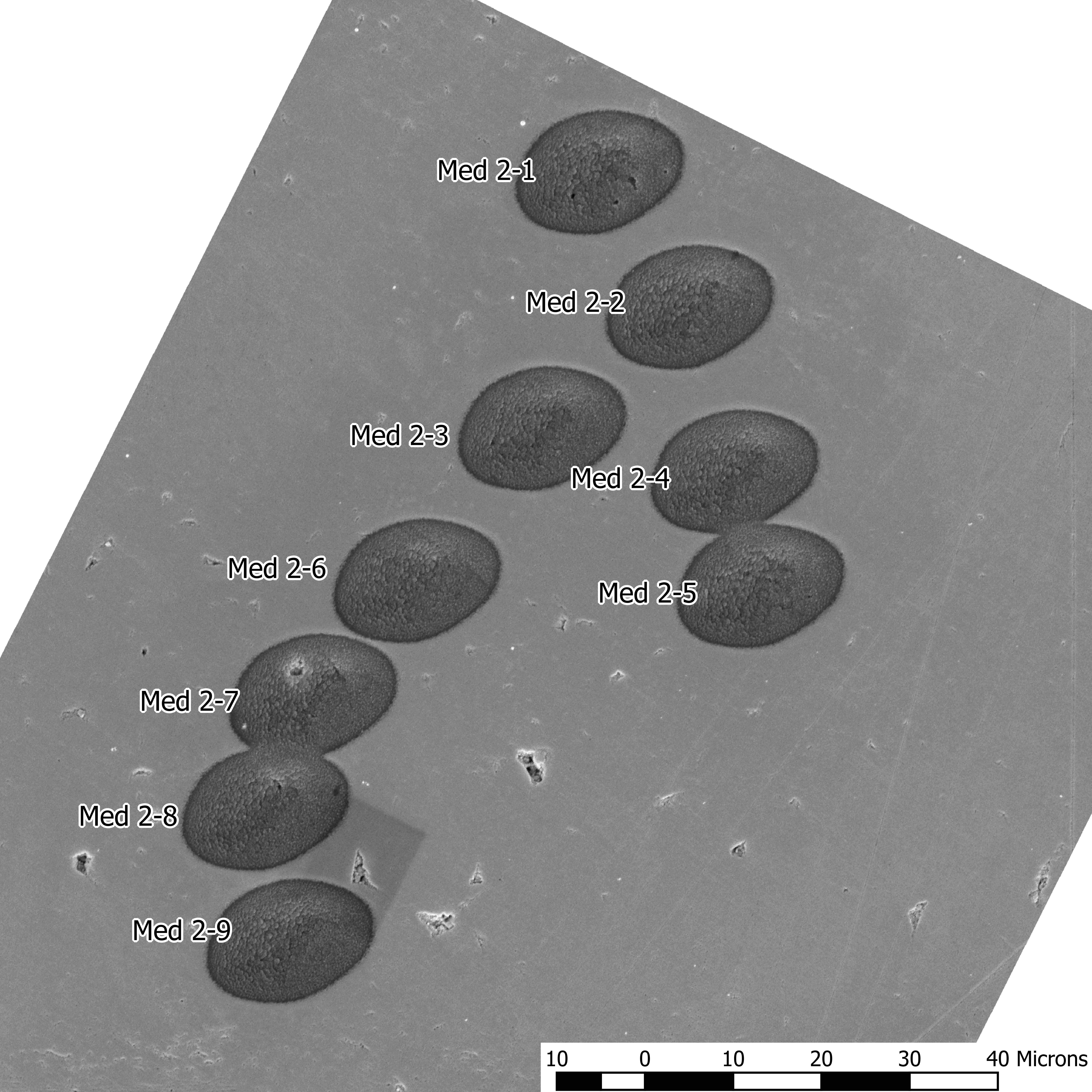
Med 2-1
Med 2-2
Med 2-3
Med 2-4
Med 2-5
Med 2-6
Med 2-7
Med 2-8
Med 2-9
10 0 10 20 30 40 Microns

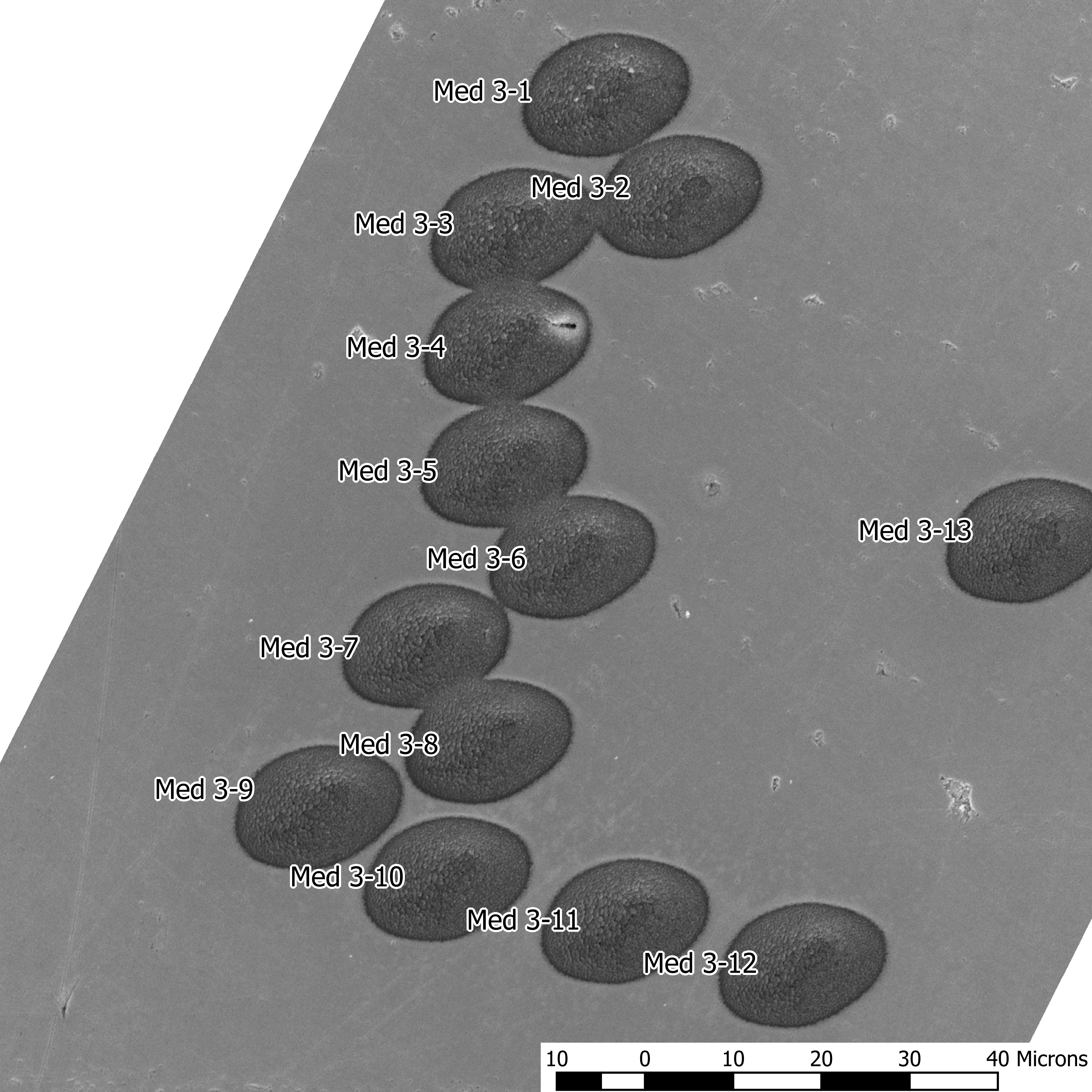
Med 3-1
Med 3-2
Med 3-3
Med 3-4
Med 3-5
Med 3-6
Med 3-7
Med 3-8
Med 3-9
Med 3-10
Med 3-11
Med 3-12
Med 3-13
10 0 10 20 30 40 Microns

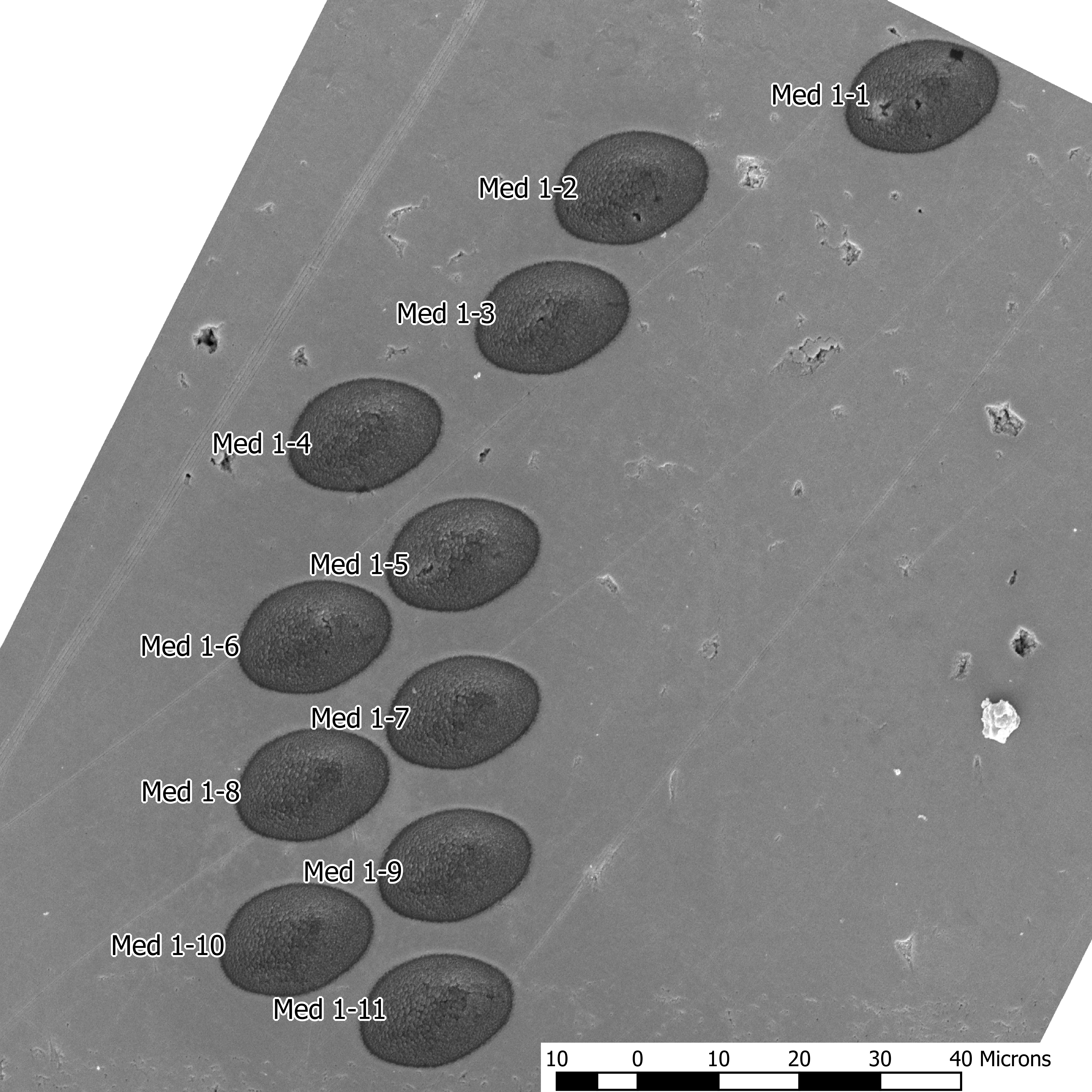

Med 1-1
Med 1-2
Med 1-3
Med 1-4
Med 1-5
Med 1-6
Med 1-7
Med 1-8
Med 1-9
Med 1-10
Med 1-11
10 0 10 20 30 40 Microns

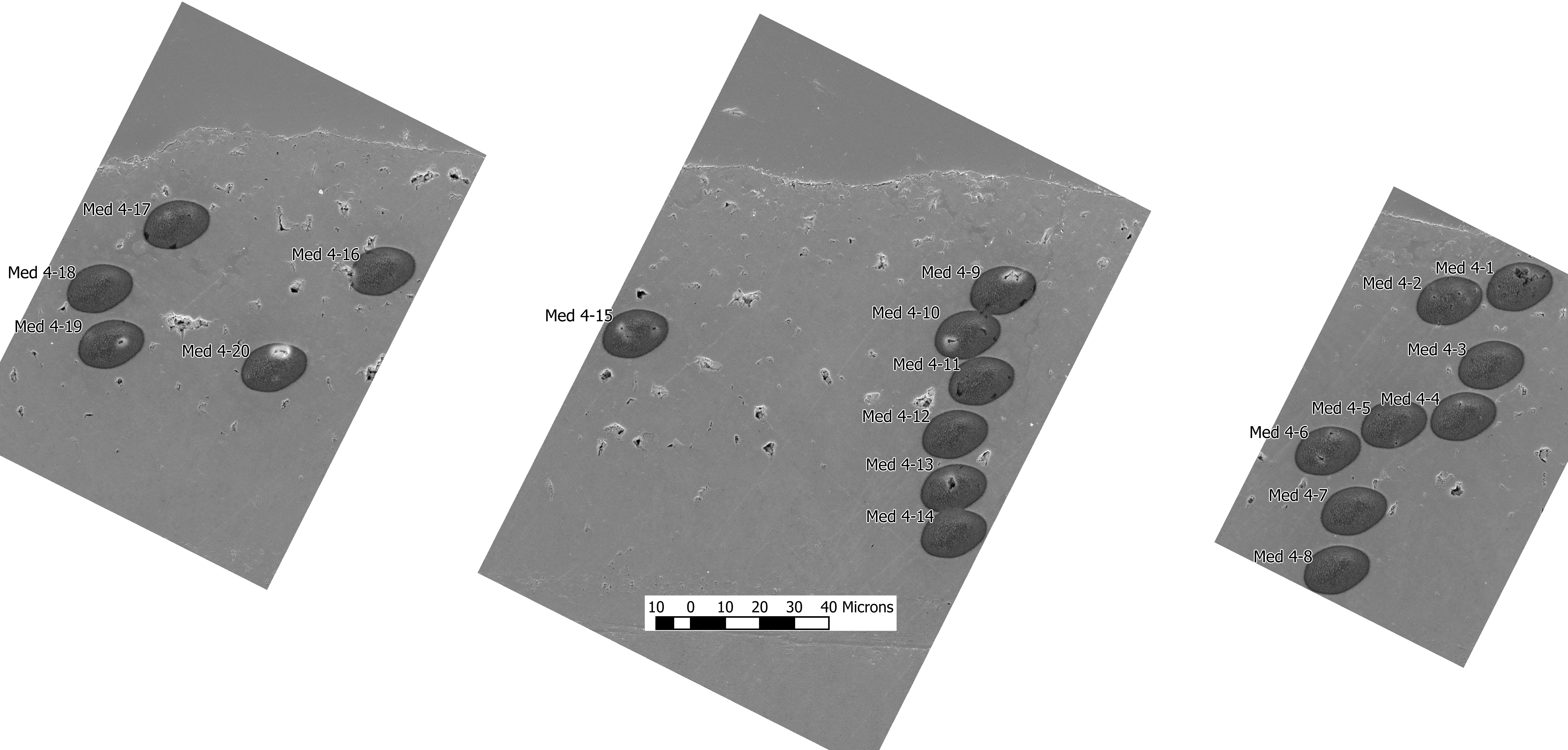
Med 4-17
Med 4-16
Med 4-18
Med 4-19
Med 4-20
Med 4-15
Med 4-9
Med 4-10
Med 4-11
Med 4-12
Med 4-13
Med 4-14
Med 4-1
Med 4-2
Med 4-3
Med 4-4
Med 4-5
Med 4-6
Med 4-7
Med 4-8
10 0 10 20 30 40 Microns
